# Antibody PEGylation in bioorthogonal pretargeting with *trans*-cyclooctene/tetrazine cycloaddition: *in vitro* and *in vivo* evaluation in colorectal cancer models

**DOI:** 10.1038/s41598-017-15051-y

**Published:** 2017-11-02

**Authors:** Aurélie Rondon, Nancy Ty, Jean-Baptiste Bequignat, Mercedes Quintana, Arnaud Briat, Tiffany Witkowski, Bernadette Bouchon, Claude Boucheix, Elisabeth Miot-Noirault, Jean-Pierre Pouget, Jean-Michel Chezal, Isabelle Navarro-Teulon, Emmanuel Moreau, Françoise Degoul

**Affiliations:** 1 Université Clermont Auvergne, INSERM U1240, Imagerie Moléculaire et Stratégies Théranostiques, F-63000 Clermont Ferrand, France; 2 Université Paris Sud, INSERM U935, Bâtiment Lavoisier, 14 Avenue Paul-Vaillant-Couturier, F-94800 Villejuif, France; 3 Institut de Recherche en Cancérologie (IRCM), INSERM U1194 – Université Montpellier – ICM, Radiobiology and Targeted Radiotherapy, F-34298 Montpellier, France

## Abstract

Bioorthogonal chemistry represents a challenging approach in pretargeted radioimmunotherapy (PRIT). We focus here on mAb modifications by grafting an increase amount of *trans*-cyclooctene (TCO) derivatives (0 to 30 equivalents with respect to mAb) bearing different polyethylene glycol (PEG) linkers between mAb and TCO (i.e. PEG_0_ (**1**), PEG_4_ (**2**) and PEG_12_ (**3**)) and assessing their functionality. We used colorectal xenograft (HT29/Ts29.2) and peritoneal carcinomatosis (A431-CEA-Luc/35A7) as tumor cells/mAbs models and fluorescent tetrazines (TZ). MALDI-TOF MS shows that grafting with **2**,**3** increases significantly the number of TCO per mAb compared with no PEG. *In vitro* immunofluorescence showed that Ts29.2 and 35A7 labeling intensity is correlated with the number of TCO when using **1**,**3** while signals reach a maximum at 10 equivalents when using **2**. Under 10 equivalents conditions, the capacity of resulting mAbs-**1–3** for antigen recognition is similar when reported per grafted TCO and comparable to mAbs without TCO. *In vivo*, on both models, pretargeting with mAbs-**2**,**3** followed by TZ injection induced a fluorescent signal two times lower than with mAbs-**1**. These findings suggest that while PEG linkers allow a better accessibility for TCO grafting, it might decrease the number of reactive TCO. In conclusion, mAb-**1** represents the best candidate for PRIT.

## Introduction

Radioimmunotherapy (RIT) consists in targeting specific antigens overexpressed by cancer cells with radiolabeled monoclonal antibodies (mAbs), ensuring selective tumor irradiation.

Some preclinical studies on colorectal cancer (CRC) and peritoneal carcinomatosis (PC) demonstrated that RIT approaches induced a significant effect alone or combined with either mAbs targeting transmembrane proteins^1–3^ or chemotherapy^4,5^. PC is a common evolution in patients with CRC and often leads to a poor survival prognosis^6^. Last years, current PC treatment, cytoreductive surgery combined with intraperitoneal chemotherapy, has notably improved the 5-year overall survival rate that still remains below 50% and strongly depends on the peritoneal carcinomatosis index (PCI) - from 44% if PCI < 6 to 7% if PCI > 17^7,8^. The limitation of current PC treatments thus supports the development of new targeted therapies such as RIT.

Although RIT presents good efficiency in the treatment of non-Hodgkin lymphomas^9^ it still remains not favoured in clinics for the treatment of solid tumors. Some practical points (cost, required radiopharmacy, arising of specific chemotherapies) limit the generalization of RIT, but the main problem lies in bone marrow toxicity. Indeed, the long half-life of radiolabeled mAbs in blood can induce hematotoxicity especially for solid tumors, which can be less sensitive as the mAbs diffuse poorly^10,11^. Thereby, some strategies including pretargeting (PT) have been developed to overcome these drawbacks. PT consists in delaying the injection of radioactivity from that of the mAb. This should allow higher activities injected leading to higher tumor dosimetry^12^. Several PT systems have been investigated since the last decades, such as streptavidin-biotin^13^ or bispecific antibodies^14^, all with some disadvantages, like immunogenicity or engineering difficulties respectively. Recently, a different PT system based on bioorthogonal chemistry features has been developed: mAbs’ lysine residues are modified by addition of *trans*-cyclooctene (TCO) moieties that can be specifically recognized by a radiolabeled tetrazine (TZ) probe^15^. This system leads to low mAb modifications by addition of small chemical groups inert towards macrobiological molecules thus decreasing immune system activation^16^. Through an inverse electron-demand [4 + 2] Diels-Alder (IEDDA) cycloaddition, TZ bind covalently to TCO with fast reaction kinetics (*k*_2_ *= *2000 M^−1^ s^−1^ methanol/water (9:1))^15^ allowing to use low concentrations of conjugated mAbs. The feasibility of IEDDA cycloaddition *in vivo* was first demonstrated by Rossin and co-workers with imaging of colon cancer xenografts^17^. Mice bearing tumors first received CC49-TCO mAb followed by ^111^In-DOTA-TZ one day later. These approaches were then developed for PET imaging on mice bearing SW1222 colorectal carcinoma xenografts by pretargeting with huA33-TCO mAb followed by ^64^Cu-DOTA-PEG_7_-TZ or ^64^Cu-SarAr-TZ 24, 48 or 120 h later, revealing high specific tumor targeting and very good contrasts^18^. Recently, pretargeting-radioimmunotherapy (PRIT) in preclinical murine models of pancreatic cancer, using an anti-CA19.9 mAb, 5B1-TCO, induced a rapid and persistent uptake in tumors from 4 h to 120 h after injection of ^177^Lu-DOTA-PEG_7_-TZ probe having a rapid clearance from non-targeted tissues^19^. This approach resulted in significant growth delay and regression of BxPC3 xenografts for a single dose injected higher to 29.6 MBq thus demonstrating the efficiency of PRIT system^19^.

This efficient strategy may however suffer from some drawbacks on the modified mAb especially the potential isomerization of functional TCO to more stable but inactive *cis*-cyclooctene (CCO)^20^, the capacity of modified antibody to react with cognate antigens, and finally the availability of TCO-mAb conjugates towards TZ. Indeed, it was recently highlighted that TCOs could possibly be masked by hydrophobic interactions with mAb^21^ and that insertion of polyethylene glycol (PEG) linkers between mAb and TCO increased more than 5-fold the ability for TZ to bind TCO moieties without altering mAb binding. Moreover, for hydrophobic fluorophores, addition of short PEG linkers has been shown to reduce aggregation of mAb conjugates^22^. Furthermore, PEGylation of drugs and proteins is well known to increase water solubility, to reduce immunogenicity and to improve both pharmacokinetics and pharmacodynamics properties^23–25^. While addition of PEG linkers on TCO-mAbs may improve their solubility and increase their tumor uptake, the rational concerning the appropriate length of PEG remains unclear.

We here address the consequences of mAbs modifications by PEG_n_-TCO_n_ on both antigen and TZ recognition to determine the best one for PRIT. First, modified mAbs harbouring a growing number of PEG_n_-TCO_n_ were compared according to grafting yield, recovery rate and *in vitro* functionality as alkylation can induce protein aggregation. Secondly, as PEGylated linkers on mAb-TCO can influence the reactivity of TCO toward TZ probes, three PEG lengths (PEG_0_-TCO (**1**), PEG_4_-TCO (**2**) and PEG_12_-TCO (**3**)) were studied both *in vitro* and *in vivo* on Ag recognition in direct targeting and pretargeting experiments.

We focused our work on two different non-internalizing mAbs, anti-TSPAN8 mAb (Ts29.2)^26^ and anti-CEA mAb (35A7)^27^, harbouring **1–3** structures. Studies were either performed on a xenograft model -HT29 cells expressing TSPAN8- and on an orthotopic peritoneal carcinomatosis model -A431-CEA-Luc expressing CEA-. Assessments were made using fluorescent TZ probes -allowing optical investigations- and represent the first step for further PRIT studies on disseminated tumors.

## Results

### Modifications of mAbs: number of TCO grafted, reactivity and stability

**Figure 1:**
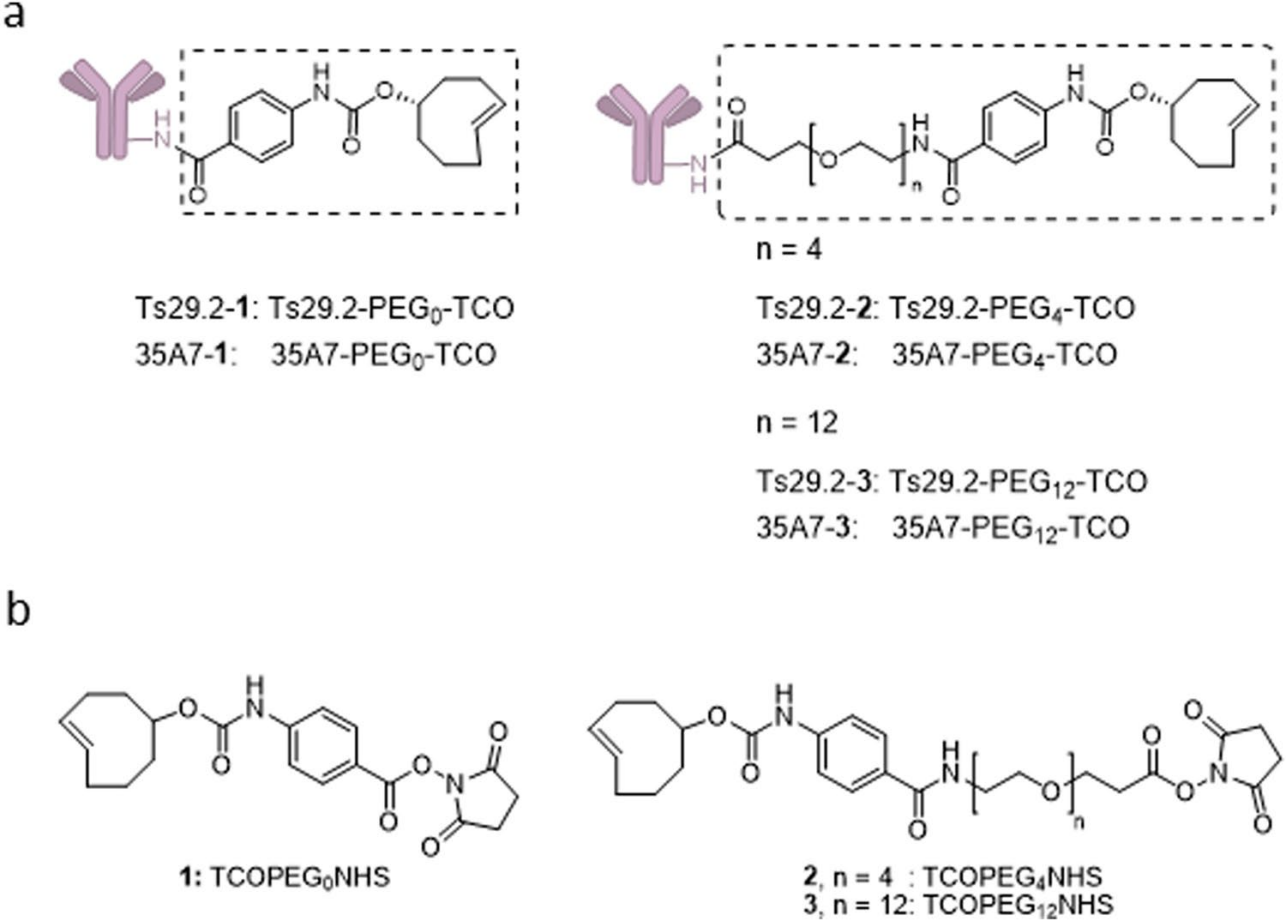
Pretargeting components. (**a**) Ts29.2 and 35A7 mAbs conjugates **1–3**. (**b**) Structures of TCO1b **1** and TCO-NHS esters derivatives **2–3**.

**Figure 2:**
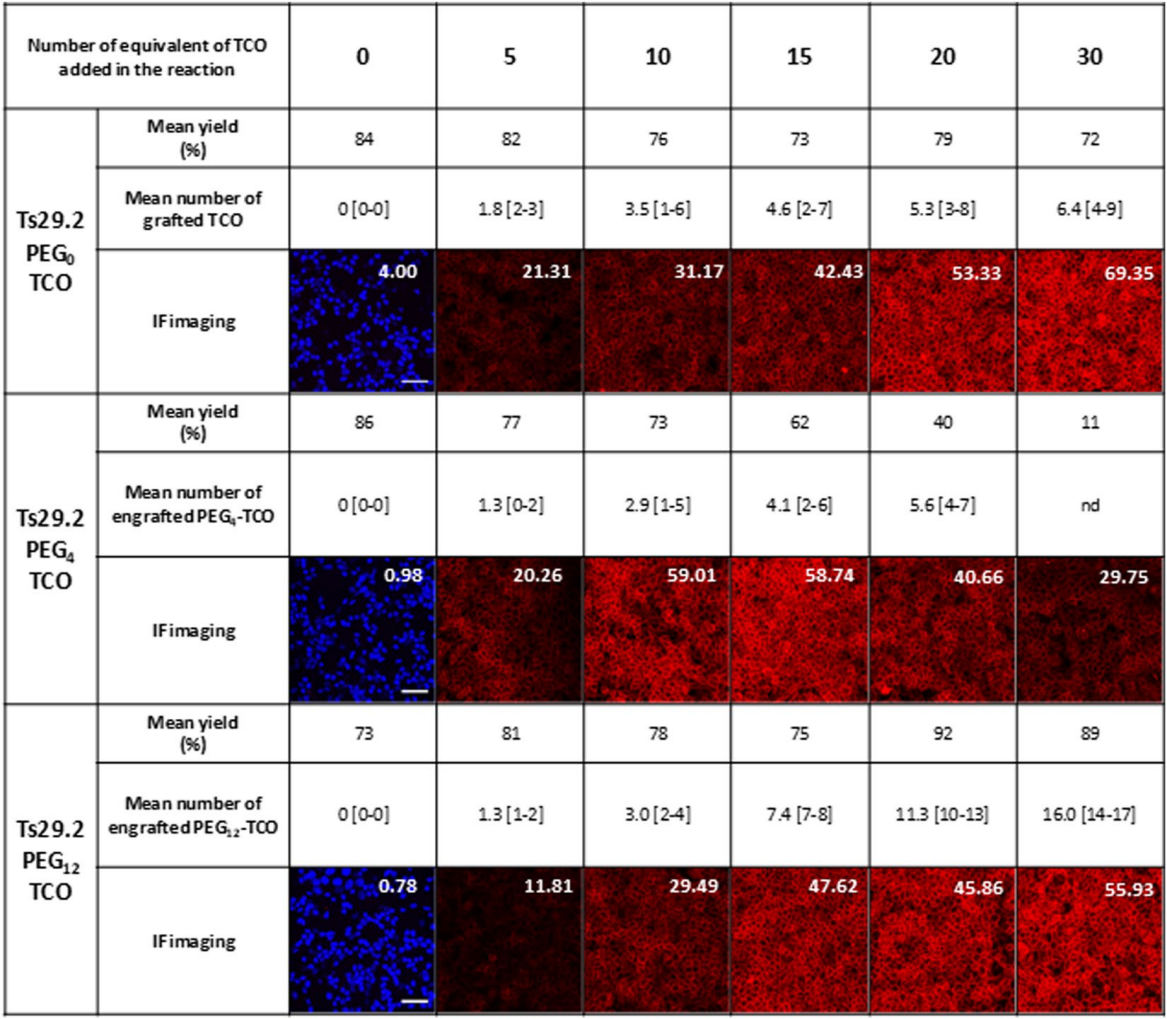
Relation between the number of TCO grafted on Ts29.2 mAbs and their functionality. Number of TCO grafted was determined by MALDI-TOF MS and is expressed as mean values [min-max], n = 3 independent experiments. Yields correspond to the mAbs recovery after grafting process. All IF imaging were made with the same settings. White numbers are mean fluorescence intensity quantified on the corresponding IF imaging. Scale bar: 50 µm.

Assessment of mAbs-**1–3** (Fig. 1a) masses (m/z) by MALDI-TOF MS allowed determining the number of TCO_n_-PEG_n_ moieties grafted on mAbs (Fig. 2 and Supp. Fig. S1). This number was similar among the experiments (n ≥ 3) and ranged from 1.3 to 16.0 for Ts29.2 and from 0.6 to 13.0 for 35A7. Interestingly, for the same number of equivalents of **1**,**3** the number of moieties grafted on mAbs increased significantly with the longest PEG spacer for both mAbs. All mAb-**1–3** were recovered with good yields after grafting and size exclusion chromatography, except Ts29.2–**2** for which there was a significant decrease when using 15, 20 and 30 equivalents of NHS ester **2** (62, 40 and 11% respectively).

We quantified the membrane fluorescence intensity with a home designed algorithm (Supp. Methods). The measured immunofluorescence intensity values for Ts29.2-**1-3** on HT29 (Fig. 2) increased with PEG0 and PEG12 according to the number of TCO grafted. In contrast, with PEG_4_ the signal reached a maximum intensity at 10 equivalents before decreasing. The same observation was made with 35A7-**1-3** (Supp. Fig. S1) on A431-CEA-Luc cells with lower signal intensity than TS29.2. According to these data, we have chosen to test mAbs modified with 10 equivalents of **1–3** for future experiments. However, as the number of **1**,**2 or 3** grafted may vary, we then normalized signal intensities by the mean of TCO engrafted. We have also assessed that mAbs-**1–3** were stable and still reactive either after freezing at −20 °C or storage at 4 °C over at least a month (Supp Fig. S2).

### Antigen recognition by modified mAbs

**Figure 3:**
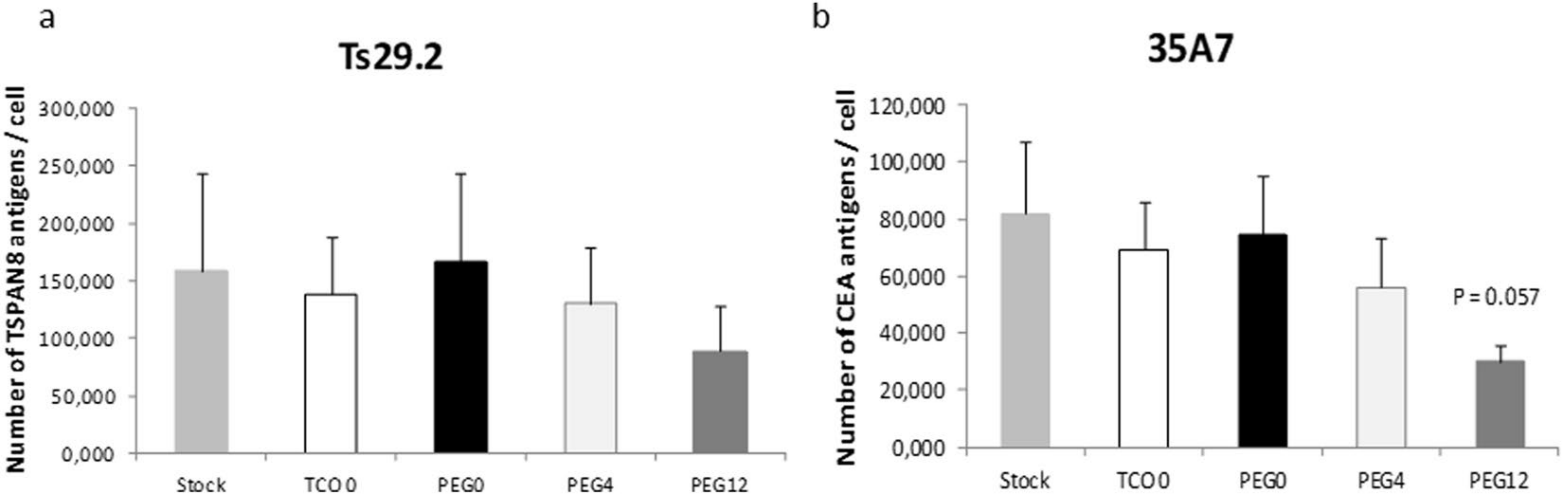
(**a–b**) Functionality of mAbs-1–3 towards their cognate antigens. Number of TSPAN8 (**a**) or CEA (**b**) antigens detected by Ts29.2 or 35A7 mAbs conjugates **1–3**. All samples were made in triplicates in three independent experiments. Statistical analyses were made using 2-paired Student T-test. P-value < 0.05 was considered significant.

In our conditions, about 150,000 TSPAN8 membrane antigens were recognized by Ts29.2 mAbs (Fig. 3a). There is no significant difference in the number of TSPAN8 antigens recognized by the unprocessed Ts29.2 (Stock), Ts29.2 processed without TCO addition (TCO 0) and the modified Ts29.2-**1-3**. Concerning the A431-CEA-Luc cells, nearly 80,000 CEA antigens were recognized by 35A7 mAbs (Fig. 3b). As obtained with Ts29.2 there was no significant difference in the number of CEA antigens recognized by 35A7-**1-3** compared to the unprocessed or unmodified one. However, there was a decrease for mAbs-**3** intensities which may be due to some aggregations already described with highly modified mAbs.

### *In vitro* functional assessment of mAbs-1-3

**Figure 4:**
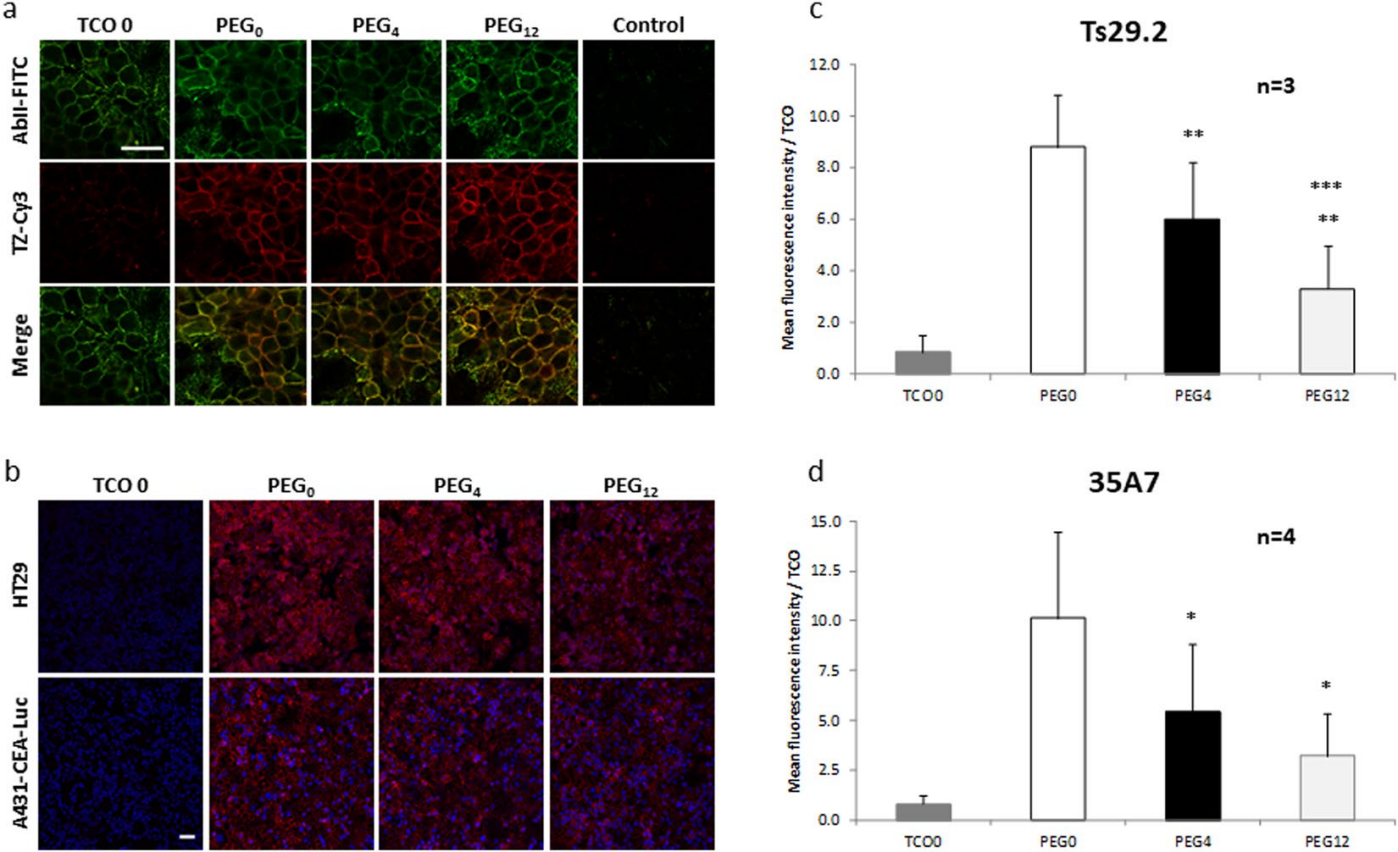
(**a**) Reliability of Ts29.2-1-3 to recognize their target and assessment of their interaction with TZ-Cy3 using confocal microscopy. HT29 cells were first incubated with 10 µg/mL of Ts29.2 processed without TCO addition (TCO 0), Ts29.2-**1** (PEG_0_), Ts29.2-**2** (PEG_4_) or Ts29.2-**3** (PEG_12_) and then with both 1/500 AbII-FITC_(495-519 nm)_ and 0.02 mM TZ-Cy3_(550-570 nm)_. Control condition corresponds to incubation without Ts29.2. Green signal corresponds to AbII-FITC labeling and red signal to TZ-Cy3 labeling. Green and red images were merged to show signal co-localization. Scale bar: 30 µm. (**b–d**) Quantification of mean fluorescence intensity per TCO for mAb-1-3. (**b**) Immunofluorescence of HT29 or A431-CEA-Luc cells incubated with mAbs-**1-3** and TZ-Cy3_(550–570 nm)_. Merged images of red: TZ-Cy3 cell membrane signal and blue: nucleus stained with DAPI. Same settings were applied for all images. Scale bar: 50 µm. Each well was imaged 3 times at random locations; all Z sections every 2 µm per field were imaged. (**c–d**) Graphs of fluorescent signal intensity obtained after ROI quantification by ImageJ software on HT29 (**c**) (n = 3 different experiments), or A431-CEA-Luc (**d**) (n = 4 different experiments). ROI were 3D-automatically computerized on the entire image and were then reported per TCO. Statistical analysis was made using two-paired Student T-test. *P < 0.05: PEG_0_ vs PEG_4_ and PEG_12_, **P < 0.01: PEG_0_ vs PEG_4_ and PEG_4_ vs PEG_12_, ***P < 0.0002: PEG_0_ vs PEG_12_.

**Figure 5:**
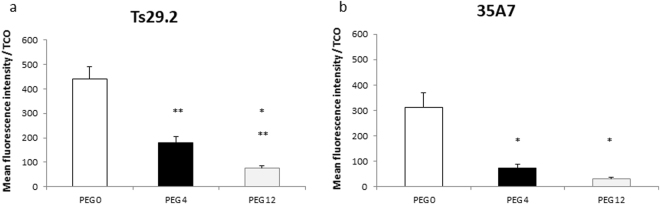
(**a–b**) Assessment of mAbs-1-3 capacity to interact with TZ-5-FAM using flow cytometry. Values are mean fluorescence intensity (MFI) ± SEM reported per the number of TCO grafted on each mAb (n = 3 independent experiments). Statistical analysis was made using two paired Student T-test. P < 0.05 was considered significant. *P < 0.05: PEG_0_ vs PEG_4_ and PEG_12_ and PEG_4_ vs PEG_12_; **P < 0.01: PEG_0_ vs PEG_4_ and PEG_12_.

Targeting of Ts29.2-**1-3** on HT29 cells (Fig. 4a) and 35A7–1–3 on A431-CEA-Luc cells with both fluorescent TZ-Cy3 and polyclonal anti-mouse Ab-FITC showed an overlay of fluorescent membrane signal (merged images), demonstrating a specific interaction between TCO groups and TZ. This comforts that modifications of mAbs-**1-3** did not alter their antigen recognition. Absence of signal with TZ-Cy3 in control cells incubated with unmodified mAbs confirmed the specificity of TCO/TZ interaction. Same observations were made with 35A7-**1-3** on A431-CEA-Luc cell model (Supp. Fig. S3). Intensity signals obtained with mAb-**2**,**3** on both cell lines showed a decrease of fluorescence compared to non-PEGylated mAbs (Fig. 4b). ROI quantification was made on the entire image and was reported per TCO (Fig. 4c, D). We observed a significant decrease of signal intensity with mAbs-**2** and mAbs-**3** compared to mAbs-**1**, for both Ts29.2 and 35A7. Results showed that the longer the PEG, the lower the signal. In addition, MFI assessed by flow cytometry using TZ-5-FAM (Fig. 5a, b) also showed a significant decrease according to the PEG linker length when normalized with the number of TCOs.

### *In vivo* functional assessments of mAbs-1-3 pretargeting

#### HT29 xenograft model

**Figure 6:**
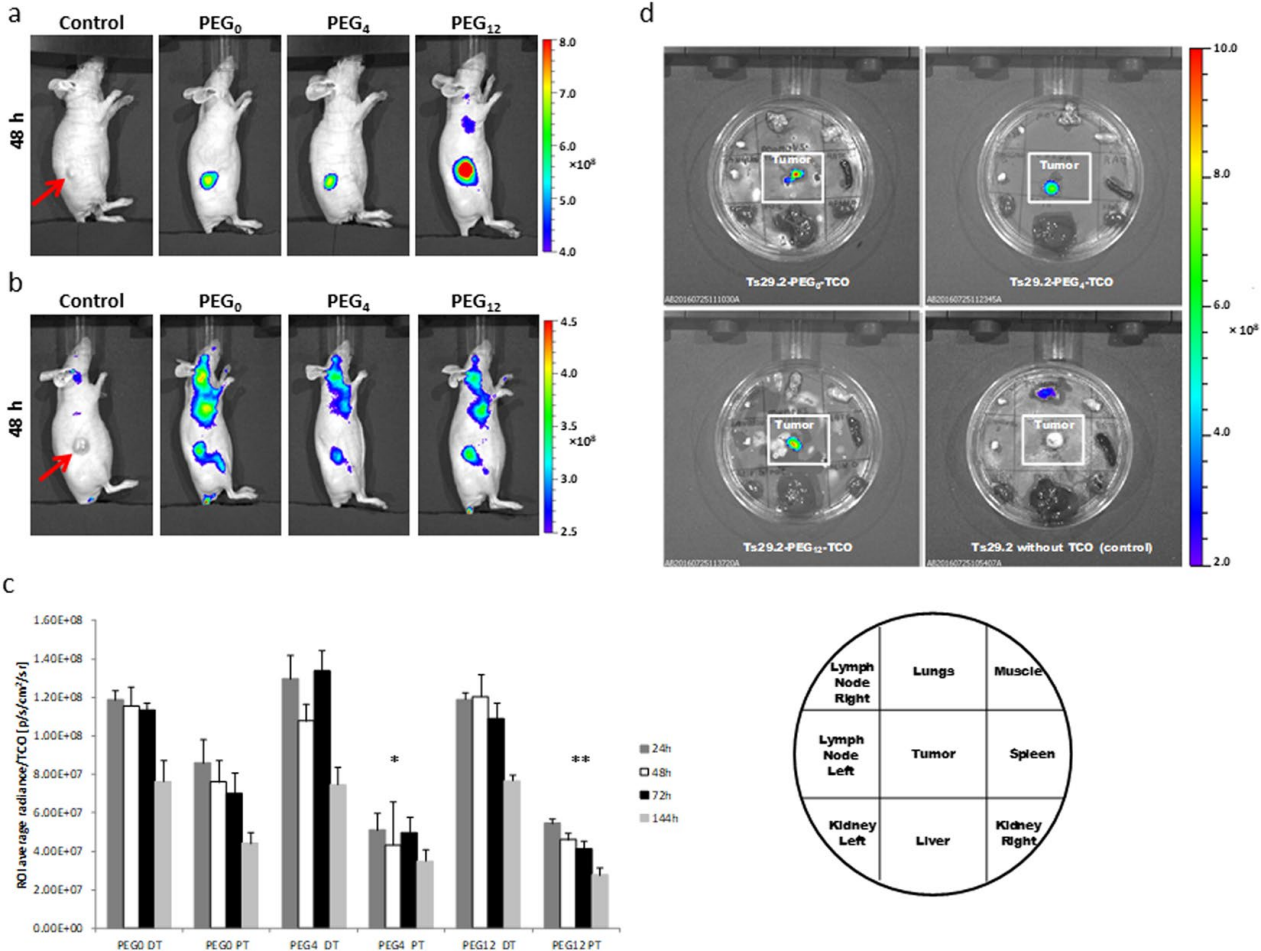
*In vivo* assessments of Ts29.2-1-3 on HT29 xenograft model. Figures represent *in vivo* imaging 48 h post TZ-Cy5 or mAb-TCO/TZ-Cy5 injections. Red arrows show the location of the tumor. (**a**) Direct targeting with Ts29.2 without TCO (Control) or Ts29.2-**1-3** after preliminary incubation 30 min with TZ-Cy5 in tube. (**b**) Pretargeting with Ts29.2 without TCO (Control) or Ts29.2-**1-3**, TZ-Cy5 was injected 24 h after mAbs. (**c**) Graph of ROI average radiance reported per the number of TCO of each mAb. Results are expressed as mean ± SEM (n = 3). Statistical analysis was made using one-way ANOVA. *P < 0.01: PEG_0_ PT vs PEG_4_ PT; **P < 0.005: PEG_0_ PT vs PEG_12_ PT. (**d**) Representative *ex vivo* imaging of tumors between other tissues. The scheme shows the repartition of the organs in the Petri box. Lymph nodes were sampled in the axillary region. Muscle corresponds to the left gastrocnemius.

Tumors were located in the right flank and were all homogeneous in size and shape. Direct targeting (DT) of modified Ts29.2 (Fig. 6a and Supp Fig. S4) showed a specific signal in tumors persistent up to 7 days post injection. ROI average radiance reported to the number of TCO grafted on mAbs (Fig. 6c) demonstrated no significant difference between PEGylated mAbs and the non-PEGylated one. In pretargeting (PT) groups (Fig. 6b) we first obtained for each mAb a significant signal in lymph nodes (LN), which progressively diminished over time after TZ-Cy5 injection. The same observation was made in the control group and in DT groups 24 h post injection (Supp Fig. S4) suggesting some venous extravasation phenomenon due to the TZ-Cy5 hydrophobic structure. After quantification of ROI signal in LN (Supp. Fig. S5) we found that the venous extravasation of TZ-Cy5 in PT groups was similar with all mAbs and therefore had no impact on the interpretation of the results.

*In vivo* fluorescent imaging of PT groups also showed a specific signal located in the tumor (Fig. 6b and Supp. Fig. S6) visible on imaging up to 3 days after TZ-Cy5 injection and quantifiable until the 7^th^ day of experimentation. ROI average radiance per TCO (Fig. 6c) demonstrated that the signal is significantly higher for Ts29.2-**1** than with PEG_4_ and PEG_12_. In addition, signal intensity in PT is two times lower than in DT, which could be attributable to the bioavailability of the hydrophobic TZ-Cy5 when injected separately from mAbs-TCO.

#### Peritoneal carcinomatosis model

**Figure 7:**
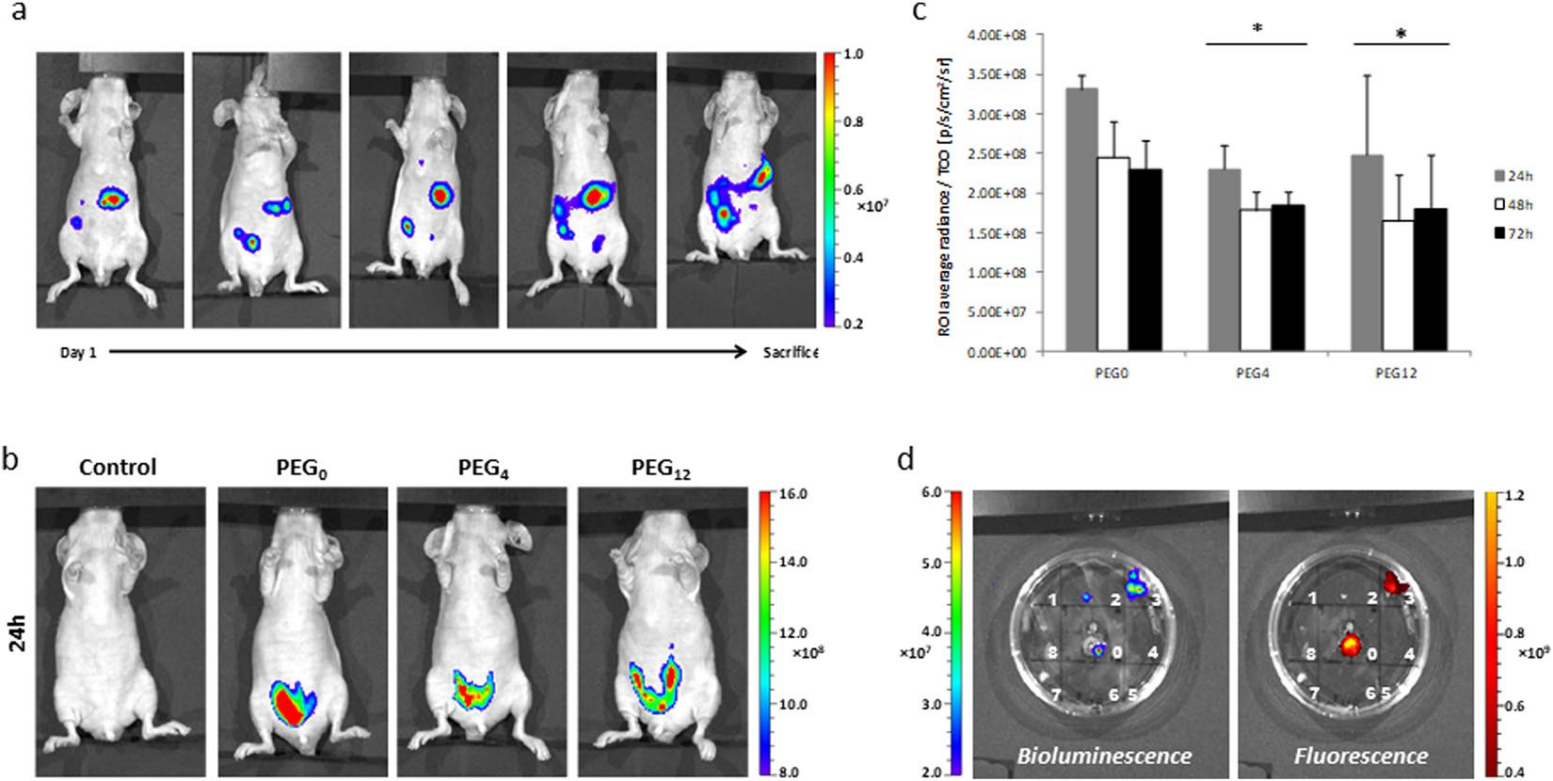
(**a**) Bioluminescence imaging of PC tumor xenografts. PC induced by IP xenografts with 1.10^6^ A431-CEA-Luc cells. Imaging was made 10 min post IP injection of 300 µL of luciferin (15 mg/mL). Representative mouse imaging from day 1 post cell engraft to sacrifice. (**b**–**d**) 35A7-1-3 pretargeting on PC tumors. (**b**) Pretargeting with 35A7-**1**-**3**. Figure represents fluorescent *in vivo* imaging 24 h post IP injection of TZ-Cy5. Imaging for 48 h and 72 h after IP injection of TZ-Cy5 are in supplementary data S8. (**c**) *In vivo* ROI average radiance of the entire peritoneal cavity, reported to the number of TCO of each modified mAb. Statistics were made using one-way ANOVA. *P < 0.05: PEG_0_ vs PEG_4_ and PEG_12_. (**d**) *Ex vivo* bioluminescent and fluorescent imaging of PC tumors. White numbers represent the 9 main peritoneal regions, as reported by Klaver *et al*.^36^.

Bioluminescence imaging during 14 days demonstrated that A431-CEA-Luc cells have gradually invaded the entire peritoneal cavity, thereby validating PC model (Fig. 7a). Mice necropsy allowed scoring the peritoneal carcinomatosis index (PCI) (Supp. Fig. S7), with a mean value around 8.9 ± 0.6 (TCO_0_: 9.0 ± 0; PEG_0_: 9.3 ± 0.9; PEG_4_: 9.3 ± 0.9; PEG_12_: 8.0 ± 0.7). Homogeneity of PCIs ensured a good repeatability of PC xenografts among the different groups, consolidating the obtained results. *In vivo* fluorescent imaging of PT of 35A7 modified mAbs 24 h, 48 h and 72 h after TZ-Cy5 labeling resulted in a specific signal in PC tumors (Fig. 7b and Supp. Fig. S8). In addition, quantification of ROI average radiance per TCO on the entire peritoneal cavity (Fig. 7c) demonstrated a significant higher fluorescent signal with 35A7-**1** compared to 35A7-**2**,**3** as obtained with Ts29.2 modified mAbs on HT29 model. Representative *ex vivo* imaging of PC tumors (Fig. 7d) assessed successively by bioluminescence and fluorescence showed an overlay of signals thus confirming the specific targeting of tumor antigens by the different modified 35A7 mAbs as observed using *in vivo* imaging.

## Discussion

The aim of this study was to evaluate the effects of mAb modifications on both antigen recognition and their ability to be recognized following binding. These modifications were introduced randomly on lysine residues presents all along the two IgG chains. This should therefore modify their capacity to recognize the targeted protein or to be recognized by antibodies or ligands.

First, we found that addition of PEG spacers on mAbs-TCO enhanced the number of moieties grafted on mAbs, especially for the longest PEG with significant higher values for both Ts29.2 and 35A7. One explanation might be the steric hindrance caused by TCO1b **1** which could hamper the accessibility to lysines while the TCO-PEG_n_-NHS alkyl chain structure could reduce this effect between adjacent groups in the folded protein. The yields of 35A7-**1-3** conjugates were stable while those of Ts29.2-**1**,**3** generally ranged at 70–90%. Surprisingly, yield of Ts29.2-**2** fell around 10% suggesting an aggregation of mAbs. It would be interesting to determine precisely on which lysine activated esters **1**, **2** and **3** are grafted in order to assess if that would influence mAbs functionality, contributing to the knowledge of grafting mechanisms. This question will be explored in further experiments.

*In vitro* evaluation using quantitative flow cytometry demonstrated on both models that purification process and mAbs’ modifications by addition of **1–3** do not alter their ability to recognize cell membrane antigens. In both immunofluorescence and CMF experiments PEGylated mAbs presented signal intensities significantly lower than the non-PEGylated one, especially for the longest PEG. This suggests that the interaction between TCO and TZ was altered by PEGylation. In contrast, Rahim and his team demonstrated *in vitro* that 90% of TCOs grafted on mAb were unreactive due to hydrophobic interactions with mAb, a phenomenon which could be overcome by increasing the distance between TCO and mAb through addition of several PEG units (PEG_4_ and PEG_24_), therefore demonstrating the longer the PEG the higher the signal^21^. This discrepancy can be explained by the difference in the mAb/TCO structures used and quantifications based on the global signal (for Rahim and his team) compared with normalized intensity to TCO (our study). However, addition of PEG linkers between mAb and TCO (PEG_12_) was suggested to increase CCO isomer formation^20^ supporting our observations.

Near infrared (NIR) fluorescence imaging on mice bearing human HT29 xenograft showed a specific signal localized in tumors which confirmed the ability of TZ-Cy5 to bind TCO directly *in vivo* as previously reported with radiolabeled TZ probes^17,19,28,29^. The high signal in axillary LN -regardless of the mAb tested- after TZ-Cy5 IV injection can be explained by extravasations in lymphatic system due to the lipophilic properties of Cy5-TZ conjugates leading to a poor solubility. On A431-CEA-Luc model indeed there was no TZ-Cy5 signal located in LN after IP injection of the TZ probe highlighting the importance of the injection mode. To our knowledge, none of the other imaging or PRIT studies made using the TCO/TZ cycloaddition detected any signal in LN. This confirms that the LN signal in our NIR fluorescence imaging is not specific and was due to the organic cyanine fluorophore, not to the TZ itself.

Comparison between DT and PT experiments demonstrated a two times lower signal for PT, which can be caused by TZ availability. Interestingly, this was reported in another pretargeting approach of LS-174T colon xenografts imaging with anti-CEA x anti-hapten mAb using the TF2 dock and lock system^30^. Biodistribution studies highlights that PT induced a lower tumor uptake and resulted in specific tumors targeting and minimal toxicity in bone marrow compared to DT. However, this PT system caused some nephrotoxicity which would imply to limit the dose injected for therapy in clinic. Nephrotoxicity was also observed in other PRIT strategies using the avidin/biotin system on colon PC tumors with a dual probe^99m^Tc-HYNIC-lys(Cy5.5)-PEG4-biotin for SPECT/CT and *ex vivo* NIR fluorescence imaging^31^. The high % of ID in kidneys observed in this PT approach can be acceptable for imaging but is pejorative for RIT. In contrast, in our models, we did not notice any fluorescence in vital organs (except the LN) thus reinforcing the safety of bioorthogonal PRIT strategy. In addition, other studies using TCO/TZ cycloaddition with radiolabeled TZ have demonstrated a rapid renal clearance of the TZ probe in few hours and residual radiation doses in kidneys comparable with the other non-targeted organs, thus comforting our NIR fluorescence imaging results^17,19,32^.

Some TCO moieties could be deactivated *in vivo* through isomerization to the unreactive *cis*-cyclooctene (CCO) isomer due to its interactions with copper-containing proteins^20,33^. Shortening the distance between mAb and TCOs was proposed and shown an increase of the steric hindrance which impeded TCOs to react with serum protein-bound copper^20^. We thus compared DT and PT using mAbs-**1–3** with the aim to determine the *in vivo* influence of the biological medium on antigen recognition and TZ interaction. Considering that the number of TCO grafted on mAbs was variable between **1**, **2** and **3** we reported the signal intensities per the mean number of TCO as we previously made for *in vitro* studies. Thereby, in PT, when normalized, PEGylation of mAbs led, in both *in vivo* models, to a diminution of the signal intensity. In addition, in DT experiments on HT29 model, we found that there was no significant difference between PEGylated and non-PEGylated Ts29.2. In those groups mAbs-**1–3** were first incubated 10 min with TZ-Cy5 and then injected in mice. Considering the fact that signal intensity is identical for the three mAbs we could confirm that PEGylation did not alter the antigen recognition. Without normalizing by TCO number (Supp. Fig. S10), the DT signal radiance significantly increased with PEG length especially with PEG_12_. The PT signal intensity also increased with **3** compared to **1** but without reaching statistical significance.

We thus demonstrated in *in vivo* pretargeting experiments that with or without normalizing the signal intensity by the number of TCO, the addition of PEG linkers should decrease the interaction between TCO moieties and TZ. Indeed, ligation between TCO and TZ leads irreversibly to a covalent cycloadduct^15^ and when incubated in saline the interaction between the two entities was complete and stable. Indeed, stability studies demonstrated that isomerization of grafted TCO moieties into the *cis* derivative is limited in the interval of time. This highlights that functional TCOs could be deactivated *in vivo* by isomerization to the unreactive CCO when they are distant from the mAb and/or that the alkyl chain could be folded against mAb and impede the access of the TZ^21^.

For our applications, PEGylation of mAbs-TCO thus do not represent an interest as it did not improve the interaction with TZ. We now planned to test the influence of PEGylation on the physicochemical properties, reactivity and stability of the TZ probes. Indeed, TZ-DOTA is a lipophilic molecule and addition of PEG linkers between DOTA chelator macrocycle and TZ should improve its solubility, thus allowing accelerating renal clearance without altering the ability of the radioligand to bind TCO-mAbs^19^.

In conclusion, we optimized an efficient process for addition of TCO and PEG-TCO on mAb by adding moieties containing few *trans/cis*-isomerization and with a reproducible number of TCO grafted per mAb. We highlighted the importance of the TCO’s number engrafted on mAbs as it can modify the interaction with TZ or antigens through *in vitro* and *in vivo* experiments. Finally, mAb-**1** containing no PEG linker represents the best candidate for next bioorthogonal PRIT experiments on disseminated tumors in peritoneal cavity. To our knowledge, this is the first demonstration of TCO/TZ IEDDA pretargeting system on PC model using NIR fluorescence imaging. The high specificity of the TCO/TZ interaction and the safety of organic dyes could enable this system to be applied in a first step for imaging guiding cytoreductive surgery of PC thus helping the surgeon to locate precisely small size tumors. In a second step, the high tumor detection we observed suggests that with appropriated therapeutic radioisotopes, PRIT could represents an alternative to conventional PC therapies.

## Methods

### Cell lines and antibodies

HT29 cell line, colon adenocarcinoma expressing TSPAN8, were provided by Dr. C. Boucheix (Inserm, Villejuif, France) as the non-internalizing murine anti-TSPAN8 monoclonal antibody (mAb) Ts29.2 (IgG2b)^34^. The cell line was maintained in Dulbecco’s Modified Eagle Medium supplemented with 10% foetal serum, 1% gentamycin and was incubated at 37 °C with 5% CO_2_ in humidified environment.

A431-CEA-Luc cell line, epithelial colon carcinoma transfected with constructs encoding both CEA and luciferase, were provided by Dr. J-P. Pouget and Dr. I. Navarro-Teulon (IRCM, Inserm, Montpellier, France) as well as the non-internalizing murine anti-CEA mAb 35A7 (IgG1)^27^. The cell line was cultured in Dulbecco’s Modified Eagle F12 Medium supplemented with 10% foetal serum, 1% penicillin/streptomycin, 1% geneticin, 1% hygromycin and was incubated at 37 °C with 5% CO_2_ in humidified environment.

### Synthesis of pretargeting components

Procedure detailed in Supplementary methods.

### mAb modifications

Procedure detailed in Supplementary methods.

### Determination of the number of 1,2 and 3 moieties per mAb

For the range experiments 100 µg of mAbs were functionalized with different equivalents of TCO and the corresponding mean numbers of TCO grafted determined using MALDI-TOF MS were reported in Fig. 2 (Ts29.2) and Supplementary Figure 2 (35A7). For *in vitro* and *in vivo* studies 500 µg of mAbs were used for TCO grafting at 10 equivalents of PEG_n_-TCO. The mean number of moieties grafted per mAb was the following: Ts29.2-1 (3.3 PEG_0_-TCO), Ts29.2-2 (4.2 PEG_4_-TCO), Ts29.2-3 (5.2 PEG_12_-TCO); 35A7-1 (2.1 PEG_0_-TCO), 35A7-2 (3.3 PEG_4_-TCO) and 35A7–3 (4.3 PEG_12_-TCO). The procedure for MALDI-TOF MS analysis is detailed in Supplementary methods and a typical quantification is reported in Supplementary Figure S9.

### mAb-1-3 stability

Procedure detailed in Supplementary methods.

### Tetrazines

For the different experiments three fluorescent TZ probes were purchased at JenaBioscience, Germany, namely: 3-(*p*-benzylamino)-1,2,4,5-tetrazine-5-Fluorescein (TZ-5-FAM) absorbance/emission lengths 492/517 nm, molecular weight (MW) 545.50 g/mol, 3-(*p*-benzylamino)-1,2,4,5-tetrazine-cyanine3 (TZ-Cy3) absorbance/emission at 550/570 nm, MW 908.07 g/mol and 3-(*p*-benzylamino)-1-2-4-5-tetrazine-cyanine 5 (TZ-Cy5) absorbance/emission at 649/670 nm, MW 826.00 g/mol. Those different fluorophores do not alter the ability of TZ to bind TCO moieties due to the high affinity and specificity of TZ with TCO and due to the ability of organic dyes to not interfere with biological function. In addition, in the same experiment we always compared mAbs labeled with the same fluorophore-TZ -i.e the most adapted to obtain significant signal detection according to laser capacity of the device we used- in order to avoid an introduction of any bias into the experiments.

### Flow cytometry

#### Determination of antigen level on cell surface

The number of TSPAN8 and CEA antigens respectively present on HT29 and A431-CEA-Luc cells’ surface was quantified using an *in vitro* kit assays (CellQuant Calibrator®, Biocytex, France) and a flow analyser (BD-LSRII flow cytometer with FACSDiva Software (BD Biosciences)). All experiments were made according to the manufacturer instructions and in triplicates. The statistical significance was tested with a PLSD Fisher test and considered to be significant when p < 0.05.

#### Assessment of the interaction between TCO and TZ

After trypsinization 1.10^6^ HT29 or A431-CEA-Luc cells were incubated 30 min at 4 °C with 0.03 nmol of Ts29.2-**1-3** or 35A7-**1-3** then washed in Dulbecco’s phosphate buffered saline (DPBS), centrifuged 5 min at 461 g and supernatants were removed to avoid unspecific ligation. Cells were finally incubated 30 min at 4 °C in dark with 10–14 equivalent of TZ-5-FAM with respect to the number of TCO or with 1/1000 donkey anti-mouse antibody labeled with AlexaFluor 488 (ThermoFisher Scientific, France). After washing and centrifugation cells pellets were resuspended in 1 mL DPBS and samples were analysed using flow analyser.

### Immunofluorescence assays

1.10^5^ HT29 or A431-CEA-Luc cells were incubated in 8 well labtek chambers (Sigma Aldrich, France) beforehand coated 1 h at RT at 5 µg/cm^2^ with rat tail collagen I (Corning, USA). Before tests, cell layers were incubated 30 min with DPBS-BSA 5% at RT and then 1h30 at 37 °C / 5% CO_2_ with 0.03 nmol Ts29.2-**1-3** or 35A7-**1-3** diluted in DPBS-BSA 2.5%. In a second step, cells were incubated 45 min at 37 °C / 5% CO_2_ with 1/1000 donkey anti-mouse labeled with Cyanine 3 (Jackson ImmunoResearch, USA) diluted in DPBS-BSA 2.5% or 10–14 equivalent of TZ-Cy3 with respect to the number of TCO diluted in DPBS-BSA 2.5% - DMSO 0.5%. Cells were then fixed with 10% formalin (Sigma Aldrich, France). Chambers were finally removed from the labteks and blades were mounted in vectashield-DAPI mounting medium then observed using a confocal imager (Leica SPE, ICCF platform, Clermont-Ferrand, France). Region of interest (ROI) was quantified on the entire image using ImageJ software. Three fields per well, all Z-sections every 2 µm, were randomly imaged and quantified with an automated 3D-strategy. Imaging settings and quantification procedure were detailed in supplementary methods.

### Animals

This investigation is conforms to the *Guide for the Care and Use of Laboratory Animals published by the US National Institutes of Health* (NIH Publication n°85–23, revised 1996). All experiments were made in accordance with the relevant guidelines and regulations and were approved by both the local Ethic committee of Clermont-Ferrand (CEMEAA n°002) and French Ministry of Education and Research (approval n°5103-2016042010209100). Twenty four female mice (Nude NRMI Foxn1^nu^/Foxn1^nu^) from Janvier Labs, (Le Genest-Saint-Isles, France) and 12 female mice (Nude NMRI Nu/Nu) from Charles River (L’Arbresle, France) were used for colon adenocarcinoma and peritoneal carcinomatosis models, respectively. Mice (5 weeks old, median weight 22 g) were housed in standard conditions (n = 5 per cage on ventilated racks, temperature 21–24 °C, 60% humidity, 12 h light/ 12 h dark cycle) with free access to standard food and water.

Intravenous (IV) injections were all made in lateral tail vein of vigil mice. *In vivo* imaging was made under general anesthesia using isoflurane gas 2.5 per cent and 2:3 oxygen. Euthanasia was made by cervical dislocation after isoflurane gas overdose. Near infrared (NIR) fluorescence imaging was made using an IVIS fluorescent imager (IVIA platform, Clermont-Ferrand, France) at 640–680 nm wavelengths. Imaging procedures were detailed in supplementary method.

### Colon adenocarcinoma model

#### Experimental design

24 female mice anesthetized with 2% isoflurane gas were subcutaneously (SC) xenografted with 3.10^6^/100 µL HT29 cells in the right flank. Then, mice were randomly divided into 8 groups (n = 3 per group). In four groups, mice were both IV injected with 0.3 nmol of Ts29.2-**1-3** or without TCO (control group) and 4–8 equivalents of TZ-Cy5 with respect to TCO. Antibodies and TZ-Cy5 were previously incubated 30 min at RT in eppendorf tube. Those groups correspond to the direct targeting groups. The four other groups were first IV injected with 0.3 nmol of Ts29.2-**1-3** or without TCO (control group) followed 24 h later by IV injection of 4–8 equivalents of TZ-Cy5 with respect to TCO, corresponding to the pretargeting groups. Antibodies and TZ-Cy5 dilutions were made in saline solution and saline with DMSO 0.5% respectively.

#### *In vivo* imaging

In both DT and PT conditions mice were imaged 2 h, 4 h and 6 h after TZ-Cy5 injections and once a day during 7 days. Mice were sacrificed at day 7.

### Peritoneal carcinomatosis model

#### Experimental design

12 female mice were intraperitoneally (IP) xenografted with 1.10^6^/250 µL A431-CEA-Luc cells. Tumor growth was followed using bioluminescence imaging through IP injections of 15 mg/mL of luciferin two times per week during 11 days. Then, mice were randomly allocated to the different groups (n = 3 per group: control, PEG_0_, PEG_4_ and PEG_12_) and were first IV injected with 0.3 nmol of 35A7 without TCO (Control) or 35A7**-1-3** followed 24 h later by IP injection of 4–8 equivalents of TZ-Cy5 with respect to TCO number. Dilutions of all mAbs and TZ-Cy5 were in saline solution and saline solution-DMSO 0.5%, respectively.

#### PCI determination

Peritoneal carcinomatosis index (PCI) is a gold-standard reference quantifying the number of peritoneal tumors ranging from 1 to 39 indicating the degree of severity^35^. We determined PCI using the sum of the score adapted for rodent by Klaver and co-workers^36^. Briefly, mice abdomen was divided into 13 regions and tumors observed in each region were scored semiquantitatively according to 0: no macroscopic lesions; 1: limited lesions from 1 to 2 mm; 2: moderate lesions from 2 to 4 mm and 3: abundant lesions (more than 10 sites or lesions over 4 mm) (Supplementary Figure S8).

#### Mice imaging

For all mice, bioluminescence imaging was made 2 h after TZ-Cy5 injection and 4 days later, 10 min after IP luciferin injection. In all groups, fluorescence imaging was made 2 h and 4 h after TZ-Cy5 injection and daily during 4 days. At day 4, mice were sacrificed and tumors were imaged by bioluminescence and fluorescence successively.

### Statistical analysis

Statistical analysis was performed using XLSTAT 2012 software. Continuous data were expressed with the mean and standard deviation (SEM). To compare continuous values of the different modified antibodies a one-way ANOVA or two-paired Student T-test were used. We considered p < 0.05 statistically significant.

## Electronic supplementary material

Supplementary information

## References

[1] Ychou M (2008). Adjuvant radioimmunotherapy trial with iodine-131-labeled anti-carcinoembryonic antigen monoclonal antibody F6 F(ab’)2 after resection of liver metastases from colorectal cancer. Clin. Cancer Res..

[2] Hanaoka H (2014). Fractionated radioimmunotherapy with ^90^ Y-labeled fully human anti-CEA antibody. Cancer Biother. Radiopharm..

[3] Derrien, A. *et al*. Therapeutic efficacy of alpha-RIT using a 213Bi-anti-hCD138 antibody in a mouse model of ovarian peritoneal carcinomatosis. *Front*. *Med*. **2** (2015). 10.3389/fmed.2015.00088 PMC4685172 26734610

[4] Aarts F (2007). A comparison between radioimmunotherapy and hyperthermic intraperitoneal chemotherapy for the treatment of peritoneal carcinomatosis of colonic origin in rats. Ann. Surg. Oncol..

[5] de Jong GM (2011). Experimental study of radioimmunotherapy versus chemotherapy for colorectal cancer. Br. J. Surg..

[6] Jayne DG, Fook S, Loi C, Seow-Choen F (2002). Peritoneal carcinomatosis from colorectal cancer. Br. J. Surg..

[7] Elias D (2015). Prognostic similarities and differences in optimally resected liver metastases and peritoneal metastases from colorectal cancers. Ann. Surg..

[8] Faron M (2016). Linear relationship of peritoneal cancer index and survival in patients with peritoneal metastases from colorectal cancer. Ann. Surg. Oncol..

[9] Witzig TE (1999). Phase I/II trial of IDEC-Y2B8 radioimmunotherapy for treatment of relapsed or refractory CD20^+^ B-cell non-Hodgkin’s lymphoma. J. Clin. Oncol..

[10] Navarro-Teulon I, Lozza C, Pèlegrin A, Vivès E, Pouget J-P (2013). General overview of radioimmunotherapy of solid tumors. Immunotherapy.

[11] Larson SM, Carrasquillo JA, Cheung N-KV, Press OW (2015). Radioimmunotherapy of human tumours. Nat. Rev. Cancer.

[12] Chatal J-F, Hoefnagel CA (1999). Radionuclide therapy. The Lancet.

[13] Hnatowich DJ, Virzi F, Rusckowski M (1987). Investigations of avidin and biotin for imaging applications. J. Nucl. Med. Off. Publ. Soc. Nucl. Med..

[14] Sharkey RM, Rossi EA, McBride WJ, Chang C-H, Goldenberg DM (2010). Recombinant bispecific monoclonal antibodies prepared by the dock-and-lock strategy for pretargeted radioimmunotherapy. Semin. Nucl. Med..

[15] Blackman ML, Royzen M, Fox JM (2008). Tetrazine ligation: fast bioconjugation based on inverse-electron-demand Diels−Alder reactivity. J. Am. Chem. Soc..

[16] Sletten EM, Bertozzi CR (2009). Bioorthogonal chemistry: fishing for selectivity in a sea of functionality. Angew. Chem. Int. Ed..

[17] Rossin R (2010). *In vivo* chemistry for pretargeted tumor imaging in live mice. Angew. Chem. Int. Ed..

[18] Zeglis BM (2015). Optimization of a pretargeted strategy for the PET imaging of colorectal carcinoma via the modulation of radioligand pharmacokinetics. Mol. Pharm..

[19] Houghton JL (2017). Establishment of the *in vivo* efficacy of pretargeted radioimmunotherapy utilizing inverse electron demand Diels-Alder click chemistry. Mol. Cancer Ther..

[20] Rossin R (2013). Highly reactive *trans*-cyclooctene tags with improved stability for Diels–Alder chemistry in living systems. Bioconjug. Chem..

[21] Rahim MK, Kota R, Haun JB (2015). Enhancing reactivity for bioorthogonal pretargeting by unmasking antibody-conjugated *trans*-cyclooctenes. Bioconjug. Chem..

[22] Sano K (2013). Short PEG-linkers improve the performance of targeted, activatable monoclonal antibody-indocyanine green optical imaging probes. Bioconjug. Chem..

[23] Chapman AP (2002). PEGylated antibodies and antibody fragments for improved therapy: a review. Adv. Drug Deliv. Rev..

[24] Harris JM, Chess RB (2003). Effect of PEGylation on pharmaceuticals. Nat. Rev. Drug Discov..

[25] Zhang X, Wang H, Ma Z, Wu B (2014). Effects of pharmaceutical PEGylation on drug metabolism and its clinical concerns. Expert Opin. Drug Metab. Toxicol..

[26] Maisonial-Besset, A. *et al*. Tetraspanin 8 (TSPAN 8) as a potential target for radio-immunotherapy of colorectal cancer. *Oncotarget* (2017). 10.18632/oncotarget.15787 PMC5400644 28423546

[27] Boudousq V (2010). Brief intraperitoneal radioimmunotherapy of small peritoneal carcinomatosis using high activities of noninternalizing 125I-labeled monoclonal antibodies. J. Nucl. Med..

[28] Devaraj NK, Thurber GM, Keliher EJ, Marinelli B, Weissleder R (2012). Reactive polymer enables efficient *in vivo* bioorthogonal chemistry. Proc. Natl. Acad. Sci..

[29] Reiner T, Zeglis BM (2014). The inverse electron demand Diels-Alder click reaction in radiochemistry: radiochemical applications of Diels-Alder click chemistry. J. Label. Compd. Radiopharm..

[30] Frampas E (2011). Pretargeted radioimmunotherapy of colorectal cancer metastases: models and pharmacokinetics predict influence of the physical and radiochemical properties of the radionuclide. Eur. J. Nucl. Med. Mol. Imaging.

[31] Dong C (2016). SPECT/NIRF dual modality imaging for detection of intraperitoneal colon tumor with an avidin/biotin pretargeting system. Sci. Rep..

[32] Houghton JL (2016). Pretargeted immuno-PET of pancreatic cancer: overcoming circulating antigen and internalized antibody to reduce radiation doses. J. Nucl. Med..

[33] Keinänen O (2017). Pretargeted PET imaging of *trans*-cyclooctene-modified porous silicon nanoparticles. ACS Omega.

[34] Ailane, N. *et al*. Effect of an anti-human Co-029/tspan8 mouse monoclonal antibody on tumor growth in a nude mouse model. *Front*. *Physiol*. **5** (2014). 10.3389/fphys.2014.00364 PMC4168815 25285080

[35] Jacquet, P. & Sugarbaker, P. H. *Peritoneal Carcinomatosis: Principles of Management* (ed. Sugarbaker, P. H.) **82**, 359–374 (Springer US, 1996).

[36] Klaver YLB (2010). Intraoperative hyperthermic intraperitoneal chemotherapy after cytoreductive surgery for peritoneal carcinomatosis in an experimental model. Br. J. Surg..

