# Supplementary material for: Antibody PEGylation in bioorthogonal pretargeting with trans-cyclooctene/tetrazine cycloaddition: in vitro and in vivo evaluation in colorectal cancer models
Source: Sci Rep. 2017 Nov 2;7:14918. doi: 10.1038/s41598-017-15051-y (PMC5668303; doi:10.1038/s41598-017-15051-y)
Supplement: Supplementary file 1 — Supplementary information [file 41598_2017_15051_MOESM1_ESM.pdf]

Supplementary Information

Antibody PEGylation in bioorthogonal pretargeting with *trans*-cyclooctene/tetrazine cycloaddition: *in vitro* and *in vivo* evaluation in colorectal cancer models.

Aurélie Rondon[1,3¤], Nancy Ty[1¤], Jean-Baptiste Bequignat[1], Mercedes Quintana[1], Arnaud Briat[1], Tiffany Witkowski[1], Bernadette Bouchon[1], Claude Boucheix[2], Elisabeth Miot-Noirault[1], Jean-Pierre Pouget[3], Jean-Michel Chezal[1], Isabelle Navarro-Teulon[3], Emmanuel Moreau[1¤], Françoise Degoul[1¤]*

¤ Co-authors
*Corresponding author

1: Université Clermont Auvergne, INSERM U1240, Imagerie Moléculaire et Stratégies Théranostiques, F-63000 Clermont Ferrand, France.
2: Université Paris Sud, INSERM U935, Bâtiment Lavoisier, 14 Avenue Paul-Vaillant-Couturier, F-94800 Villejuif.
3: Institut de Recherche en Cancérologie (IRCM), INSERM U1194 – Université Montpellier – ICM, Radiobiology and Targeted Radiotherapy, F-34298 Montpellier cedex 5.

***Corresponding author**: **Dr Françoise DEGOUL, PhD** UMR 1240 INSERM UCA, 58 Rue Montalembert, 63005 Clermont-Ferrand cedex; Tel: (+33)4 73 15 08 14; Fax: (+33)4 73 15 08 01;
E-mail: francoise.degoul@inserm.fr

Supplementary methods

Syntheses of pretargeting components. TCO and TCO1b 1 (**Fig.1a**) were synthesized as reported by Rossin and co-workers in 2010[1]. TCOPEG$_n$NHS derivatives **2-3** (**Fig.1a**) were synthesized according to the following general procedure. TCO 1b **1** (24 mg, 0.062 mmol) was first dissolved in anhydrous CH_2Cl_2 (1 mL). Then triethylamine (87 µL, 0.62 mmol) and a solution of PEGylated amino acids (PEG$_4$ amino acid (15-Amino-4,7,10,13-tetraoxapentadecanoic acid (CAS 663921-15-1), 35 mg, 0.093 mmol, 1.5 eq) – or PEG$_{12}$ amino acid (37-Amino-4,7,10,13,16,19,22,25,28,31,34,37-dodecaoxanonatria-contanoic acid (CAS 1415408-69-3), 113 mg, 2.5 eq) in anhydrous CH_2Cl_2 (1.5 mL) were successively added. An amount of 0.5 eq of PEGylated amino acid (PEG$_4$: 12 mg, 0,031 mmol; and PEG$_{12}$: 23 mg, 0,031 mmol) in anhydrous CH_2Cl_2 (1 mL) were added 16 h later and the reaction mixtures were stirred at RT for respectively 1 and 6 more days. Then bis(2,5-dioxopyrrolidin-1-yl)carbonate (50 mg, 0.186 mmol), triethylamine (26 µL, 0.186 mmol) and catalytic *N*,*N*-dimethylaminopyridine (1,5 mg, 0.012 mmol) were successively added and the reaction mixtures were stirred at RT for 1 h. The mixtures were successively washed with 0.5 N aqueous HCl (2.5 mL), H_2O (3 x 3 mL) and brine (6 mL) and the organic layers were dried over $MgSO_4$ and concentrated under reduced pressure.

Compound **2**: Crude product containing TCOPEG$_4$NHS **2** was purified by column chromatography on C-18 reversed phase silica gel (H_2O/CH_3CN 55/45) and furnished the desired compound as a mixture of TCO- and CCOPEG$_4$NHS (91/9; 17 mg, 43%). TCOPEG$_4$NHS **2**: ^1H NMR (500 MHz, $CDCl_3$): δ 7.75 (d, J = 9.0 Hz, 2H), 7.42 (d, J = 9.0 Hz, 2H), 6.85 (bs, 2H), 5.65-5.47 (m, 2H), 4.45 (m, 1H), 3.79 (t, J = 6.5 Hz, 2H), 3.66-3.59 (m, 16 H), 2.84 (t, J = 6.5 Hz, 2H), 2.81 (s, 4H), 2.40-1.56 (m, 10H); ^{13}C NMR (125 MHz, $CDCl_3$): δ 169.0 (q), 166.9 (q), 166.7 (q), 152.8 (q), 141.1 (q), 134.9, 133.1, 129.0 (q), 128.2, 117.7, 81.5, 70.7, 70.6 x 2, 70.5 x 2, 70.3, 69.8, 65.7, 41.1, 39.8, 38.6, 34.2, 32.5, 32.2, 31.0, 25.6; HRMS (ESI+): *m/z* 634.2980 $[M+H]^+$, Calcd for $C_{31}H_{44}O_{11}N_3$ 634.2970.

CCOPEG$_4$NHS: ^1H NMR (500 MHz, $CDCl_3$): δ 7.74 (d, J = 9.0 Hz, 2H), 7.42 (d, J = 9.0 Hz, 2H), 6.98 (bs, 1H), 6.92 (bs, 1H), 5.71-5.55 (m, 2H), 4.83 (m, 1H), 3.78 (t, J = 6.5 Hz, 2H), 3.64-3.58 (m, 16 H), 2.83 (t, J = 6.5 Hz, 2H), 2.79 (s, 4H), 2.40-1.57 (m, 10H); ^{13}C NMR (125 MHz, $CDCl_3$): δ 169.1 (q), 167.0 (q), 166.8 (q), 152.9 (q), 141.2 (q), 129.8, 129.5, 128.9 (q), 128.2, 117.7, 116.3, 77.0, 70.7, 70.6, 70.5 x 2, 70.4, 70.2, 69.9, 65.7, 39.8, 33.9, 33.8, 32.1, 25.6, 24.8, 22.3; HRMS (ESI+): *m/z* 634.2966 $[M+H]^+$, Calcd for $C_{31}H_{44}O_{11}N_3$ 634.2970.

Compound **3**: Several attempts to purify crude product containing TCOPEG$_{12}$NHS **3** (TCO/CCO 96/4, quantitative) by normal and C18-reversed-phase chromatography were undertaken and failed to provide the desired activated NHS esters which tend to massively isomerize and/or hydrolyze.

Spectroscopic data were consistent with previously reported data for this compound[2]. Crude product **3** was then coupled to mAbs as such without purification for preliminary studies.

mAb modifications. Both Ts29.2 and 35A7 mAbs were functionalized by addition of different amounts of TCO in the reaction, namely 0, 5, 10, 15, 20 or 30 equivalents of TCO for 1 equivalent of mAb. The distance between mAb and TCO was also modulated by insertion of PEGylated spacers of various lengths, i.e. mAb-**1**, mAb-**2** and mAb-**3** (**Fig1b**).

An amount of 100 µg of Ts29.2 or 35A7 mAb concentrated in DPBS was buffered with 1 M aqueous $NaHCO_3$ to adjust the pH to 9. Then, a solution of TCO1b **1** or TCO-PEG_n-NHS esters **2,3** (0, 5, 10, 15, 20 and 30 eq) dissolved in DMSO was added to the mixture (5 % DMSO final concentration). Reactions were stirred for 30 min in the dark at 4 °C and then purified on Zeba desalting columns (40 kDa MW cut-off, 0.5 mL) (Pierce Zeba™ desalting columns, Thermo Scientific). mAbs-**1-3** conjugates were kept in the dark either at -20 °C or 4 °C. All reactions were performed at least in triplicates. Stability studies were made *in vitro*, following the procedure described in supplementary methods. For *in vivo* experiments, the same grafting protocol was applied on 500 µg of mAb. Final concentration of mAbs-**1-3** conjugates was measured using a Multiskan GO microplate spectrophotometer (Fisher Scientific, France). We determined a yield of mAb recovery taking into account the starting quantity of mAb and the final one after desalting columns.

Determination of the number of 1,2 and 3 moieties per mAb. Assessments were made using MALDI-TOF MS analyzes (Voyager DE-Pro mass spectrometer, Sciex, USA). Sinapinic acid (Sigma, France) was diluted in acetonitrile/water (30/70, v/v) with 0.1 % TFA at 10 mg/mL to obtain the matrix solution. A set of three serial dilutions from 1 mg/mL of mAb-**1-3** in PBS was prepared by mixing those with the matrix solution (2/1, v/v). Acquisitions were performed in a positive linear mode and 600 shots were averaged for each spectrum. The average mAb-**1-3** molecular weight was obtained from the mass of the $[M+H]^+$ peak for the dilution set. Calibration settings corresponded to a close external mode using IgG1 (AB Sciex, USA). The number of **1-3** grafted per mAb was quantified using the molecular weight difference between the mAbs-**1-3** and the unmodified mAbs, net masses added depending on the modification (about 272, 520 and 872 Dalton for **1**, **2** and **3** respectively).

mAbs-1-3 stability. Stability of both Ts29.2-**1-3** and 35A7-**1-3** was assessed *in vitro* with TZ-5-FAM. After grafting TCO and PEG-TCO moieties mAbs were aliquoted and either frozen at -20 °C or keep at 4 °C. TCO/TZ interaction was assessed 7, 14 and 28 days after storage. 5 µg of mAbs-**1-3** was added to Laemli 4X and heated to 95 °C for 5 min.

Samples were then loaded on SDS-PAGE acrylamide gels 4-15 % (Biorad, France). After migration, gels were entirely incubated 5 min in a solution containing 0.02 mM of TZ-5-FAM (10-14 equivalents

with respect to TCO) and then rinsed under gentle shaking 15-20 min in deionized water before being imaging (G:Box, Ozyme, France). Gels were finally colored with Simplyblue™ SafeStain following the manufacturer's instructions (Thermofisher Scientific, France) and imaged with Chemidoc imager (Biorad, France). Quantification was made using both ImageJ and ImageLab software. A ratio between the fluorescence intensity and the amount of protein was made for the main mAb form to assess the reactivity of TCOs. In parallel, aliquots were analyzed by MALDI-TOF MS to determine the mean number of TCO grafted.

In vivo imaging settings

Optical images were acquired using a small animal imaging system (IVIS spectrum, Perkin Elmer, USA) and a dedicated software (Living Image 4.5 software, Perkin Elmer, USA). Acquisitions were performed using the following parameters. Bioluminescence: automatic exposure time; binning: medium; F/stop: 1; excitation filter: blocked; emission filter: open. NIR fluorescence for cyanine 5 imaging: automatic exposure time; binning: medium; F/stop: 2; excitation filter: 640 nm; emission filter: 680 nm. Images were analyzed using Living Image. Regions of interest (ROI) were drawn manually, and light was quantified as photons/seconds/cm^2/steradian. Signal was represented using inverse rainbow color. Images were processed minimally as no smoothing was applied. All comparative images were threshold at the same MAX and MIN intensities.

Confocal imaging settings

The same settings were applied for acquisitions of all quantified images. We used three lasers: 405 nm for DAPI imaging, 488 nm for FITC and 532 nm for Cyanine3. The same laser intensity was applied for all images in order to compare each mAb. Imaging resolution were 1024 x 1024 pixels. We applied a zoom = 1. An exception was made for co-localization images which are not quantified, images were in 520 x 520 pixels with a zoom of 2. An offset of -0.5 was applied for removing background noise with a gain of 950. Phase correction was equal to -36.92. Bidirectional imaging with Z-steps every 2 µm were made on the whole cell layer. Three random fields per well were imaged and quantified in three or four independent experiments.

Signal quantification on confocal imaging

To quantify the intensity of the signal localized on cell membrane we developed a 3D-automatized method on ImageJ software. Procedure steps is detailed in the following macro:

1. title0=getTitle();
2. run("Reduce Dimensionality...", "slices keep");
3. close(title0);
4. title=getTitle();
5. run("Z Project...", "projection=[Average Intensity]");
6. selectWindow(title);
7. run("Z Project...", "projection=[Max Intensity]");
8. setOption("BlackBackground", true);
9. setAutoThreshold("Huang dark");
10. run("Convert to Mask");
11. run("Options...", "iterations=4 count=4 black pad do=Open");
12. run("Measure");
13. imageCalculator("AND create", "MAX_"+title,"AVG_"+title);
14. run("Measure");
15. RID1 = getResult("RawIntDen", 0);
16. RID2 = getResult("RawIntDen", 1);
17. print(255*RID2/RID1);
18. IJ.deleteRows(0, 1);
19. close("Results");

Supplementary Figures

Number of equivalent of TCO added in the reaction		0	5	10	15	20	30
**35A7 PEG$_0$ TCO**	**Mean yield (%)**	95	89	94	95	90	91
	**Mean number of grafted TCO**	0 [0-0]	0.6 [0-1]	1.6 [1-2]	2.2 [1-3]	3.0 [2-4]	3.7 [3-4]
	**IF imaging**	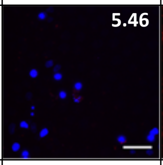	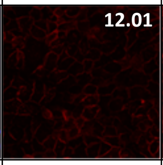	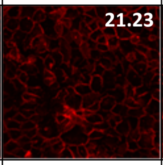	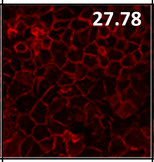	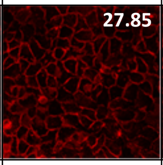	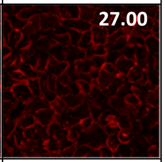
**35A7 PEG$_4$ TCO**	**Mean yield (%)**	95	95	100	94	98	93
	**Mean number of engrafted PEG$_4$-TCO**	0 [0-0]	1.7 [1-2]	3.7 [3-4]	5.7 [5-7]	7.6 [6-9]	11.6 [10-13]
	**IF imaging**	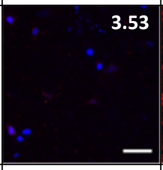	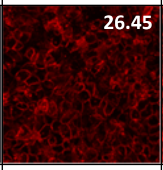	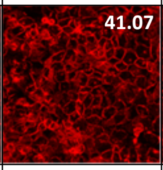	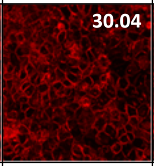	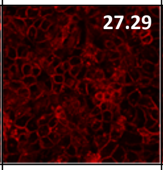	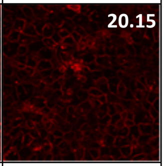
**35A7 PEG$_{12}$ TCO**	**Mean yield (%)**	95	100	96	97	92	100
	**Mean number of engrafted PEG$_{12}$-TCO**	0 [0-0]	1.2 [1.1-1.3]	2.8 [2.5-3.1]	6.3 [6-7]	8.8 [8-9]	13.0 [12-14]
	**IF imaging**	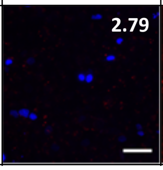	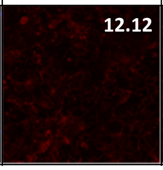	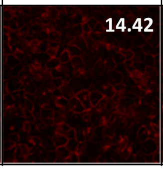	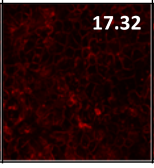	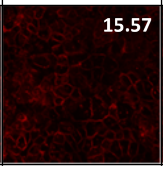	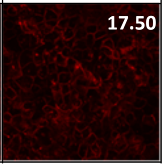

Supplementary Figure S1: Relation between the number of TCO grafted on 35A7mAbs and their functionality. Number of TCO grafted was determined by MALDI-TOF MS and is expressed as mean values [min-max], n= 3 independent experiments. All IF imaging were made with the same settings. Yields correspond to the mAb recovery after grafting process. White numbers are mean fluorescence intensity quantified on the corresponding IF imaging. Scale bar: 50 µm.

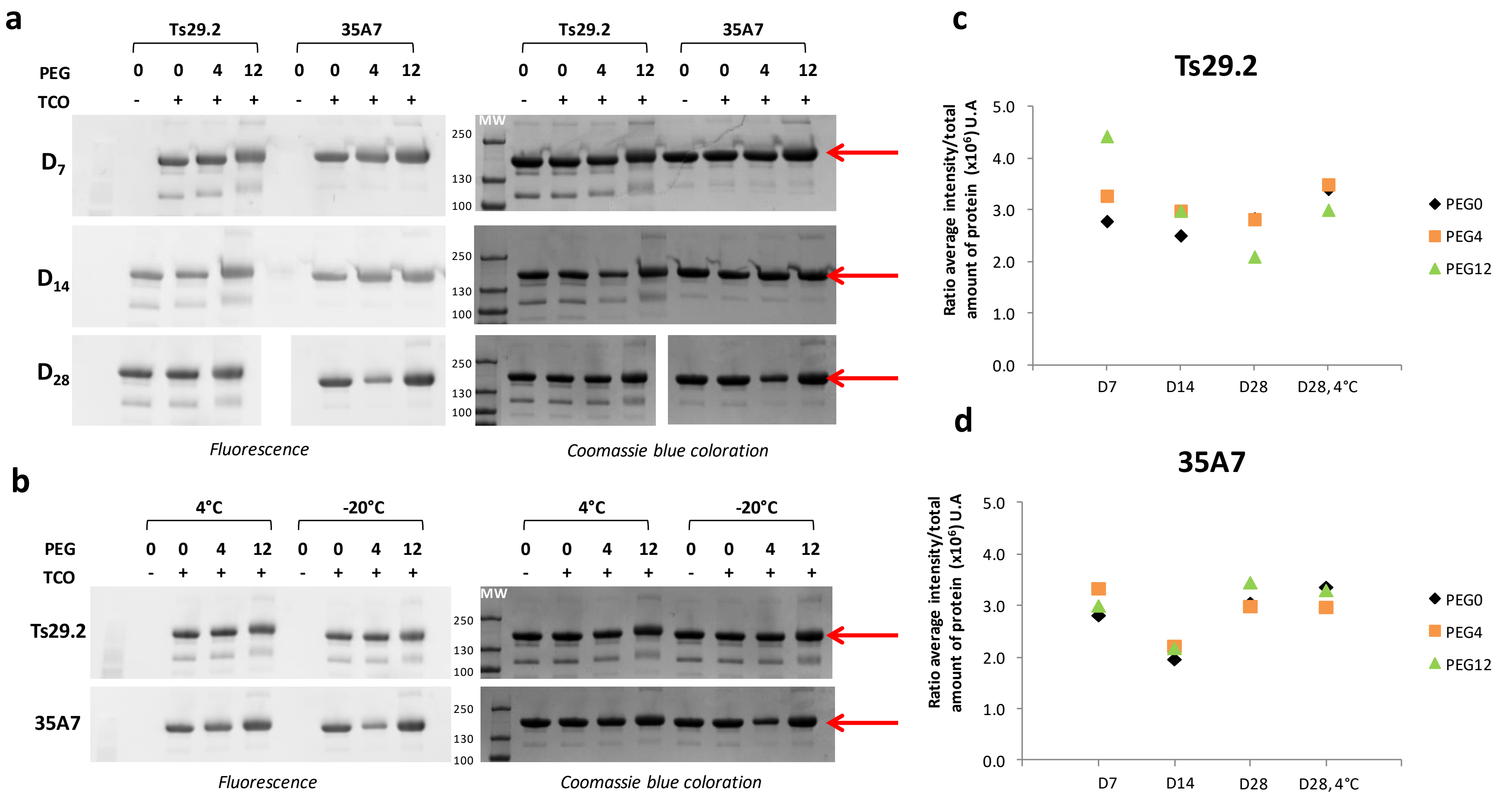

Supplementary Figure S2: Characterization of mAb-1-3 and assessment of their stability. (a) Gels' imaging in fluorescence and after Coomassie blue coloration of mAbs-**1-3** after TZ-5-FAM labeling. Samples were stored at -20°C. Stability was assessed 7, 14 and 28 days after grafting. D28 are parts from two different gels. Full gels are provided in Supplementary Figure S11. (b) Comparison at day 28, between samples stored at -20°C and 4°C. Molecular weight (MW) expressed in kDa. (c-d) Quantification of the average intensity of fluorescence reported on the total amount of protein. Quantification was made on the major band only (red arrows).

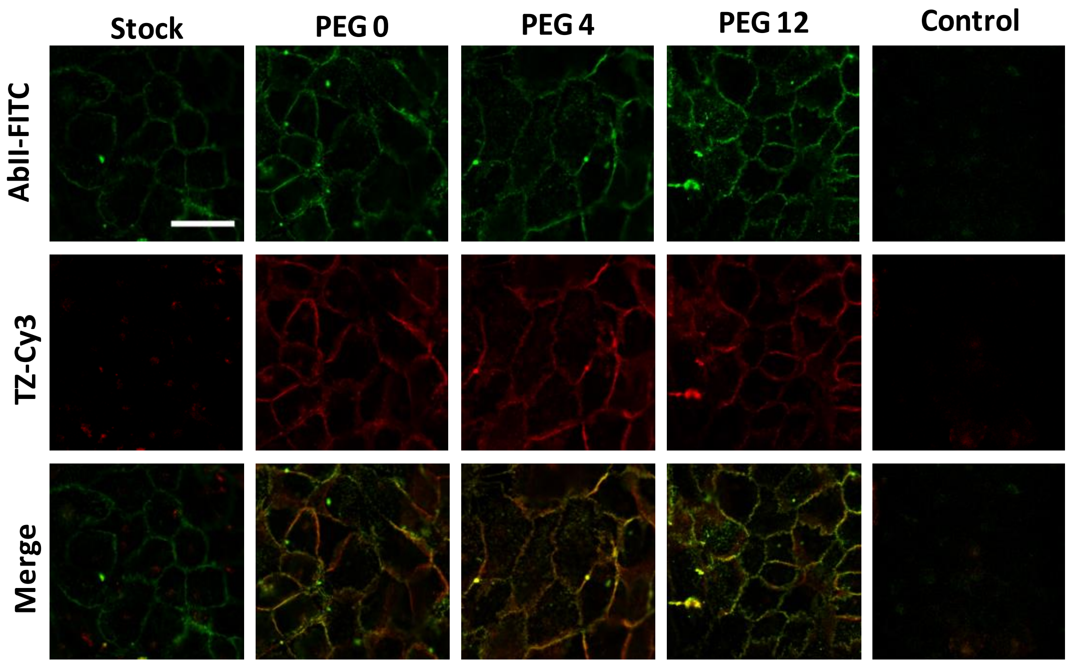

Supplementary Figure S3: Reliability of 35A7-1-3 to recognize their target and assessment of their interaction with TZ-Cy3 using confocal microscopy. A431-CEA-Luc cells first incubated with 10 µg/mL of 35A7 Stock (without TCO), 35A7-**1**, 35A7-**2** or 35A7-**3** and then incubated with both 1/500 AbII-FITC$_{(495\text{-}519nm)}$ and 0.02 mM TZ-Cy3$_{(550\text{-}570\ nm)}$. Control condition correspond to incubation without 35A7. Green signal correspond to AbII-FITC labeling and red signal to TZ-Cy3 labeling. Green and red images were merged to show signal co-localization. Scale bar: 30 µm.

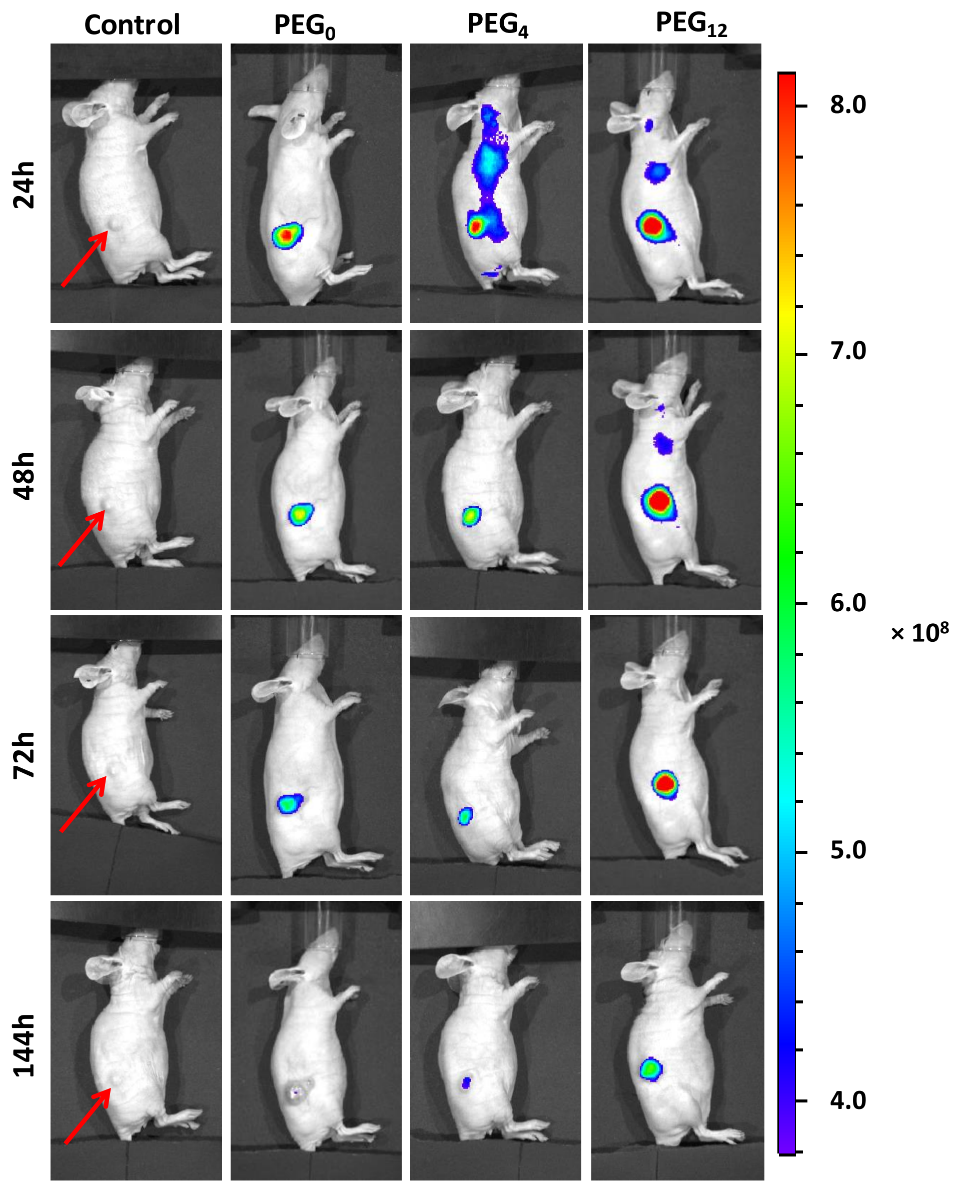

Supplementary Figure S4: ***In vivo*** **direct targeting of Ts29.2-1-3 after fluorescent TZ-Cy5 labeling on mice bearing HT29 colon xenograft.** TZ-Cy5 was injected simultaneously with modified Ts29.2 after a preliminary incubation of 30 min in tube. Figure represents *in vivo* imaging 24 h, 48 h, 72 h and 144 h after TZ-Cy5 injection. The red arrows show the location of the tumor.

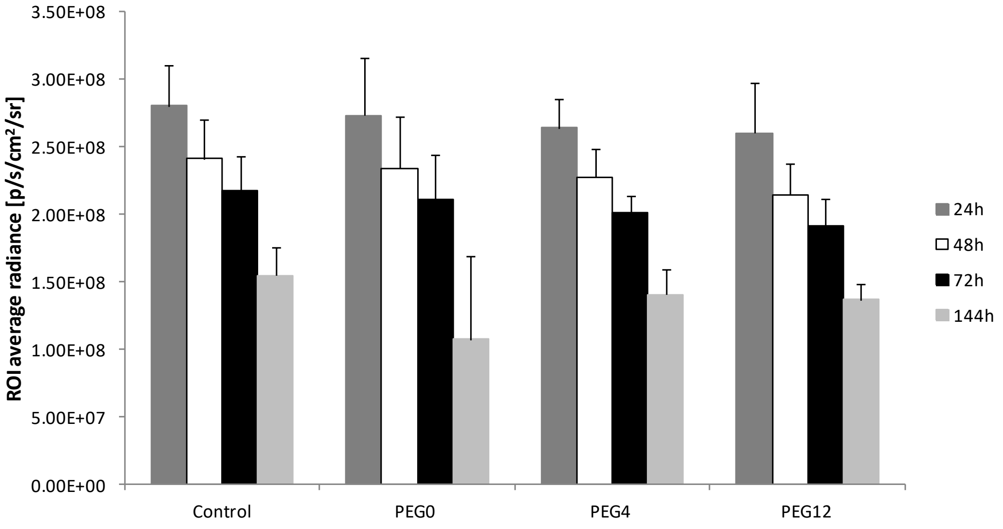

Supplementary Figure S5: *In vivo* quantification of signal located in lymph nodes in pretargeting groups in HT29 xenograft model. Graph represents ROI average radiance ± SEM (n=3). Quantification made on *in vivo* imaging at 24 h, 48 h, 72 h and 144 h post TZ-Cy5 injection.

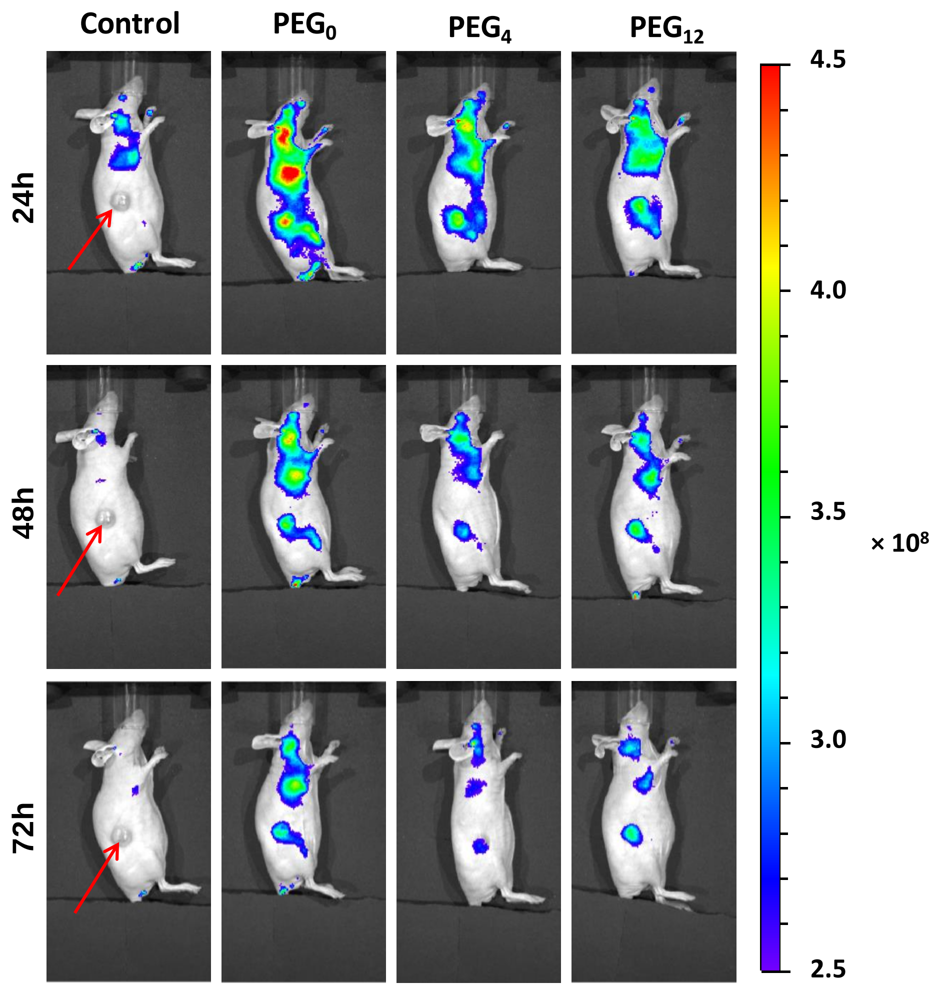

Supplementary Figure S6: *In vivo* pretargeting of Ts29.2-1-3 after fluorescent TZ-Cy5 labeling on mice bearing HT29 colon xenograft. TZ-Cy5 was injected 24 h after modified Ts29.2. Pretargeting with Ts29.2 without TCO (Control) Ts29.2-**1** (PEG_0) Ts29.2-**2** (PEG_4) or Ts29.2-**3** (PEG_{12}). Figure represents *in vivo* imaging 24 h, 48 h and 72 h after TZ-Cy5 injection. Imaging at 144 h post TZ-Cy5 injection were not shown here as signal was quantifiable but not visible when all images were treated at the same threshold. Red arrows show the location of the tumor.

Condition	Mouse	Region number													Total PCI
		0	1	2	3	4	5	6	7	8	9	10	11	12	
TCO 0	Mouse 1	3	0	1	3	0	1	0	1	0	0	0	0	0	9
	Mouse 2	0	0	0	3	3	0	1	0	2	0	0	0	0	9
	Mouse 3	0	0	1	3	1	0	1	3	0	0	0	0	0	9
PEG_0	Mouse 1	1	0	3	3	0	0	1	1	1	0	0	0	0	10
	Mouse 2	2	0	1	3	1	1	1	1	0	0	0	0	0	10
	Mouse 3	1	0	0	3	0	0	2	1	1	0	0	0	0	8
PEG_4	Mouse 1	1	0	1	3	0	0	3	2	0	0	0	0	0	10
	Mouse 2	3	0	1	3	0	0	2	0	1	0	0	0	0	10
	Mouse 3	3	0	0	3	0	0	0	0	2	0	0	0	0	8
PEG_{12}	Mouse 1	0	0	4	1	0	2	0	2	0	0	0	0	0	9
	Mouse 2	3	0	0	3	0	1	0	1	0	0	0	0	0	8
	Mouse 3	0	0	0	3	0	1	2	1	0	0	0	0	0	7

Supplementary Figure S7: Peritoneal carcinomatosis index (PCI). PCI was determined during mice necropsy, 17 days after engraftment. Each peritoneal region was attributed a score from 0 to 3. Scores were determined according to the method of Sugarbaker[3] **a**dapted on rodent by Klaver et al. [4].

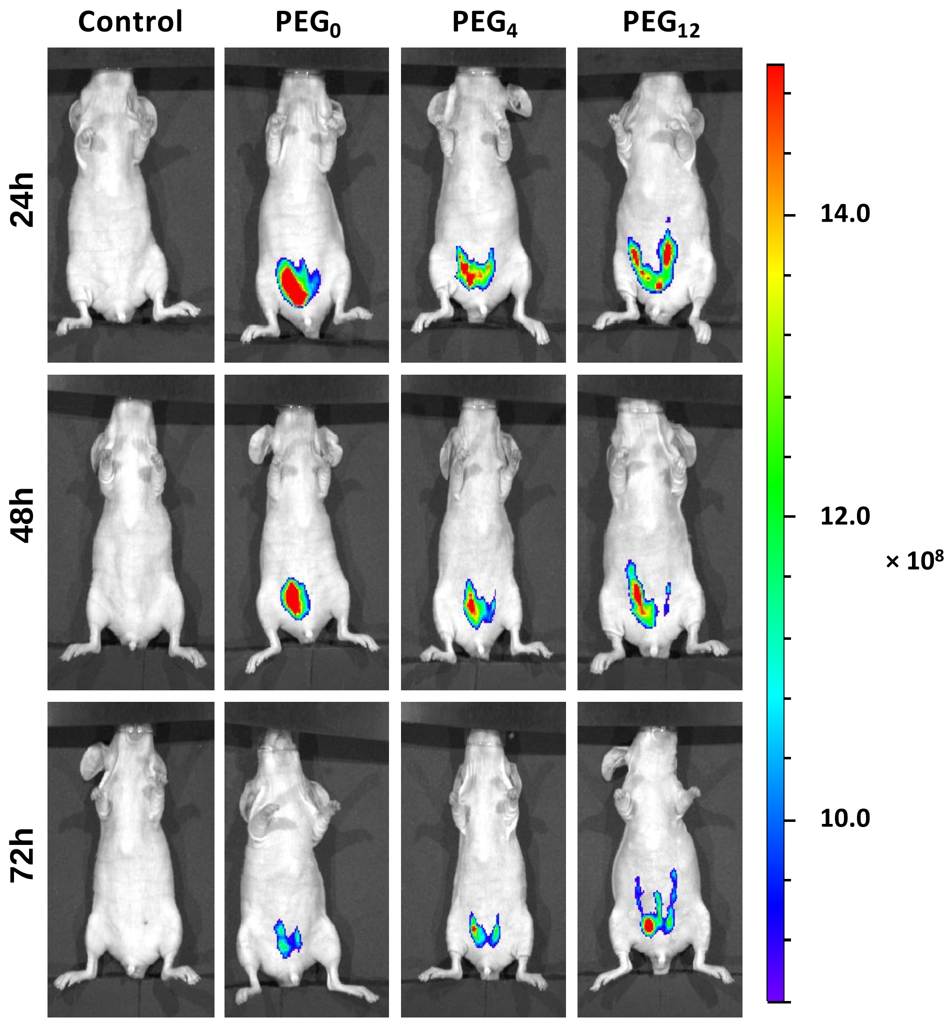

Supplementary Figure S8: Assessment of 35A7-1-3 pretargeted PC tumors after TZ-Cy5 labeling. Pretargeting with 35A7 without TCO (Control), 35A7-**1** (PEG_0), 35A7-**2** (PEG_4) or 35A7-**3** (PEG_{12}). Figure represents fluorescent *in vivo* imaging 24 h, 48 h and 72 h after IP injection of TZ-Cy5.

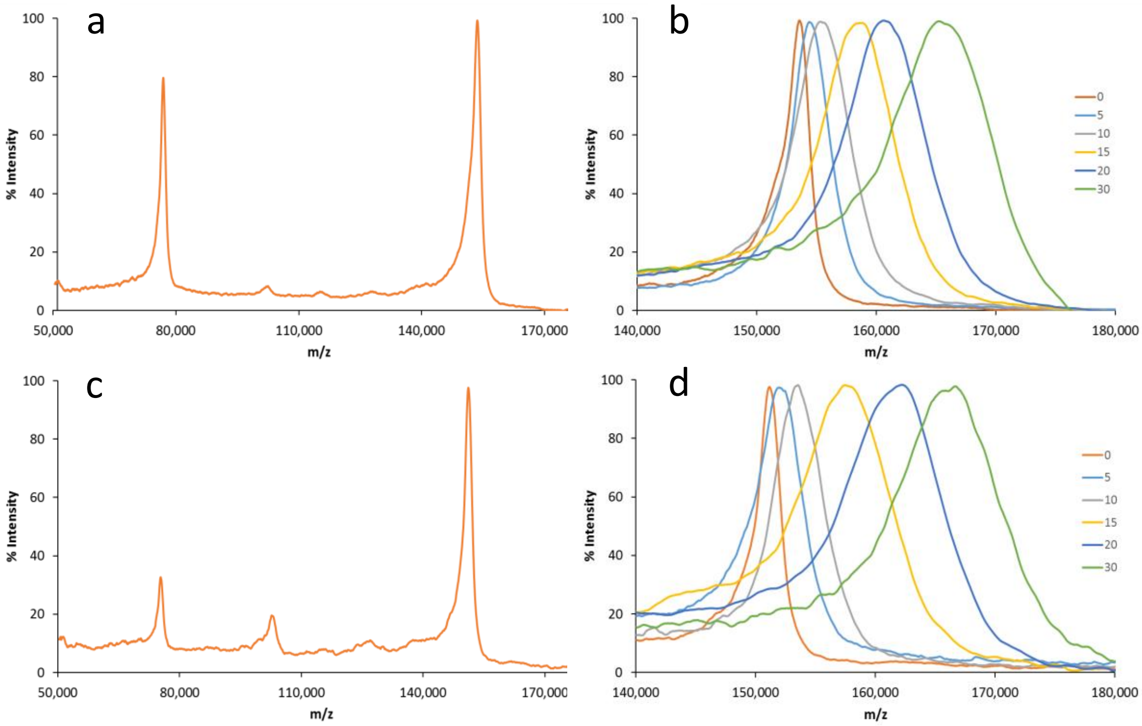

Supplementary Figure S9: MALDI-TOF mass spectrometry of 35A7 and Ts29.2 mAbs modified using different ratios of **3**. (a) Representative spectrum of the unmodified 35A7 mAb. Single protonated $[M+H]^+$ peak at ≈153,000 Dalton, double charged peak at ≈76,500 Dalton. (b) Merged representative spectra of 35A7-**3** after addition of 0 to 30 equivalents of **3**. (c) Representative spectrum of the unmodified Ts29.2 mAb. Single protonated $[M+H]^+$ peak at ≈151,000 Dalton, double charged peak at ≈75,500 Dalton. (d) Merged representative spectra of Ts29.2-**3** after addition of 0 to 30 equivalents of **3**. The number of **3** moieties bound to mAbs was determined using the shift of mass (about 872 Dalton for each **3** added).

a

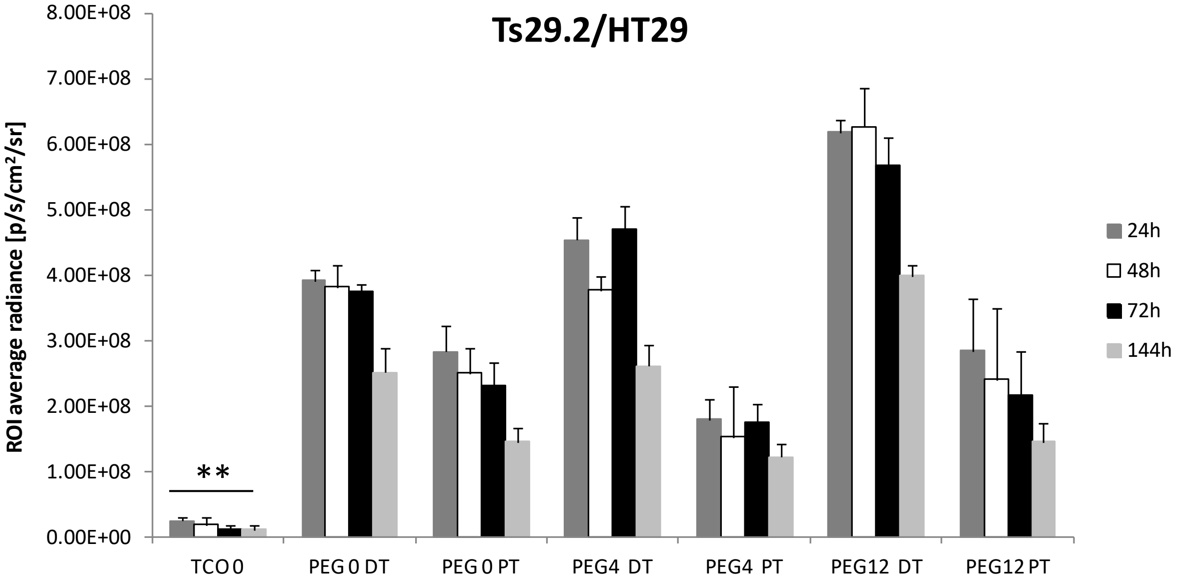

b

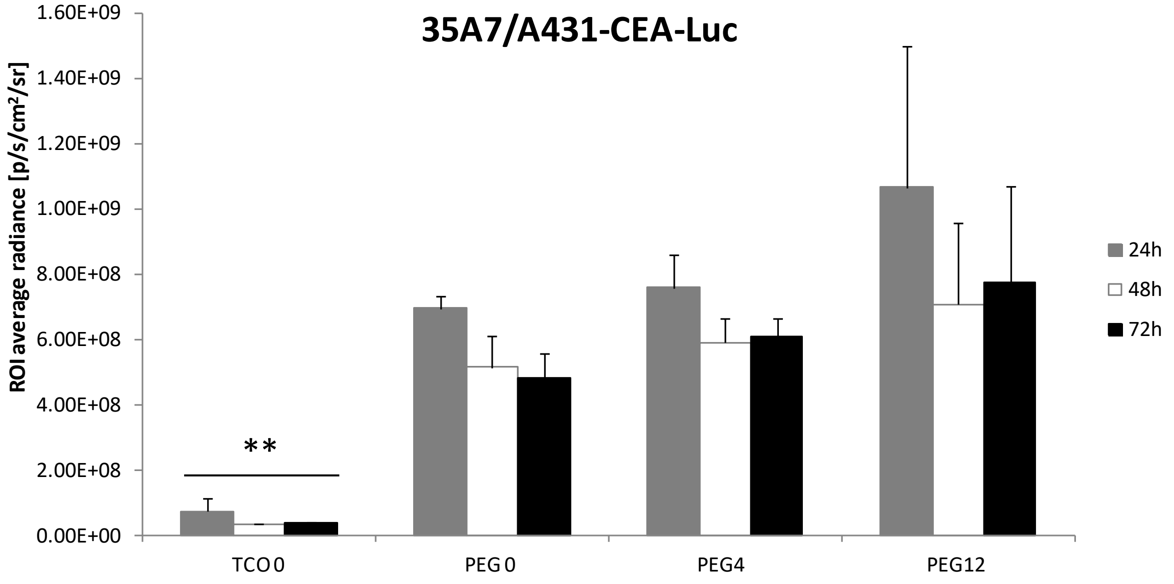

Supplementary Figure S10: ***In vivo*** **pretargeting assessments.** Graphs presenting ROI average radiance of (a) Ts29.2-**1-3** and (b) 35A7-**1-3**. DT = Direct targeting; PT = pretargeting; TCO 0 = processed control mAb containing 0 TCO. Values are expressed as mean ± SEM (n=3). Statistical analysis was made using one-way ANOVA. ** P < 0.0001: TCO 0 (all times) vs all groups.

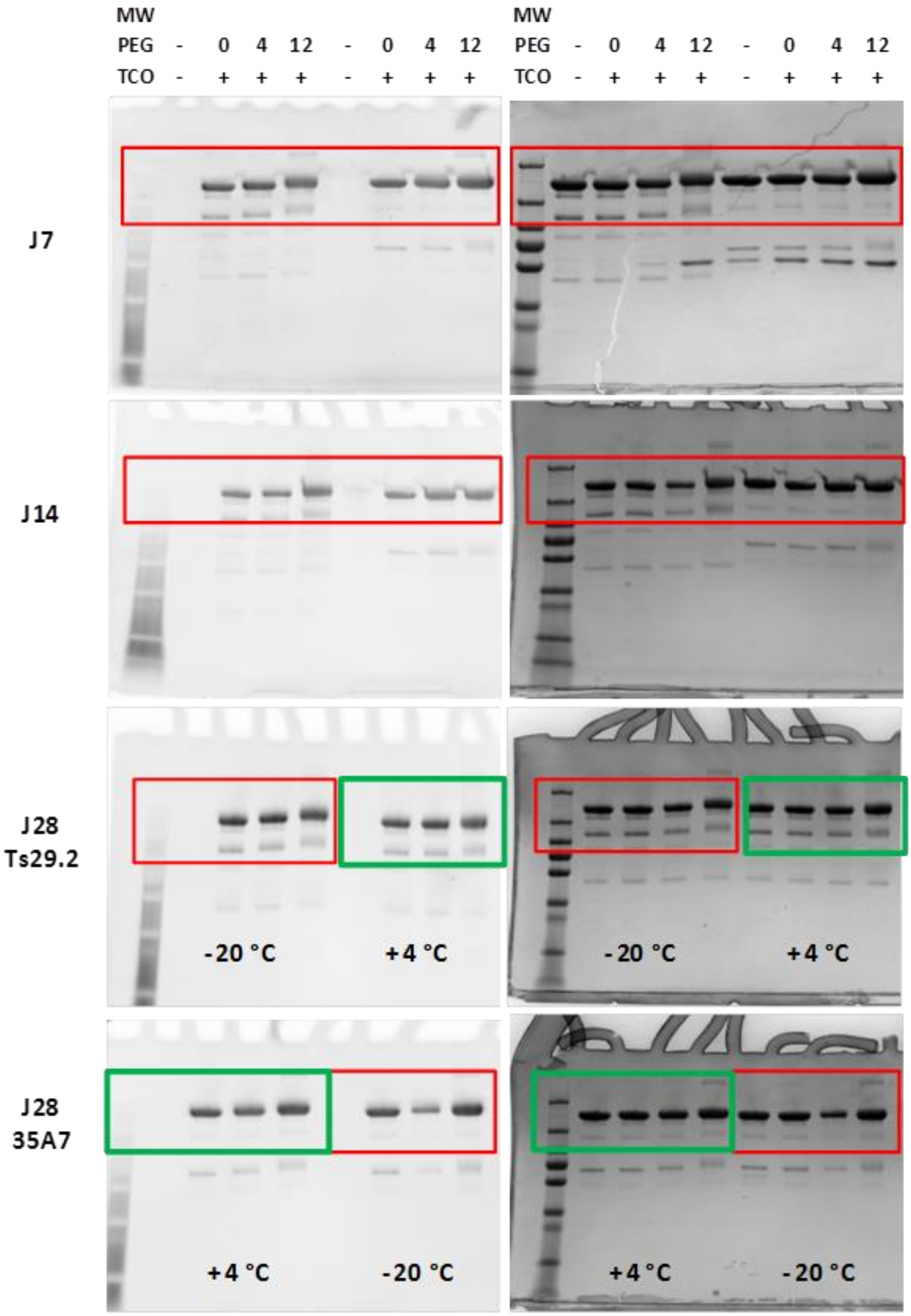

Supplementary Figure S11: **Characterization of mAbs-1-3**. Full gels from Supplementary Figure S2. Refer to Supplementary Figure S2 legend. Red (- 20 °C) and green (+ 4 °C) squares represent the cropped regions.

References:

1. Rossin, R. et al. *In vivo* chemistry for pretargeted tumor imaging in live mice. *Angewandte Chemie International Edition* **49**, 3375–3378 (2010).

2. Rossin, R. et al. Highly reactive *trans*-cyclooctene tags with improved stability for Diels–Alder chemistry in living systems. *Bioconjugate Chemistry* **24**, 1210–1217 (2013).

3. Jacquet, P. & Sugarbaker, P. H. *Peritoneal Carcinomatosis: Principles of Management* (ed. Sugarbaker, P. H.) **82**, 359–374 (Springer US, 1996).

4. Klaver, Y. L. B. et al. Intraoperative hyperthermic intraperitoneal chemotherapy after cytoreductive surgery for peritoneal carcinomatosis in an experimental model. *British Journal of Surgery* **97**, 1874–1880 (2010).
